# Process modularity, supply chain responsiveness, and moderators: The Médecins Sans Frontières response to the Covid‐19 pandemic

**DOI:** 10.1111/poms.13696

**Published:** 2022-03-03

**Authors:** Félicia Saïah, Diego Vega, Harwin de Vries, Joakim Kembro

**Affiliations:** ^1^ Hanken School of Economics HUMLOG Institute, Supply Chain and Social Responsibility Helsinki Finland; ^2^ Erasmus School of Economics Econometric Institute Erasmus Universiteit Rotterdam Rotterdam The Netherlands; ^3^ Department of Industrial Management and Logistics Lund University Lund Sweden

**Keywords:** Covid‐19 pandemic, humanitarian supply chain, moderators, process modularity, supply chain responsiveness

## Abstract

The unprecedented scale of the Covid‐19 pandemic has been a challenge for health supply chains around the world. Many international humanitarian organizations have had to ensure the continuity of their already complex development programs, while addressing their supply chain disruptions linked to the pandemic. Process modularity has frequently been advocated as a strategy to mitigate such disruptions, although empirical evidence regarding its impact on supply chain responsiveness and what moderates this impact is scarce. This exploratory research uses supply chain data analysis, qualitative content analysis, interviews, and a three‐round Delphi study to investigate how Doctors without Borders (Médecins Sans Frontières; MSF) and its 151 missions employed process modularity during the Covid‐19 pandemic. Our results show that despite severe disruptions, process modularity—based on a modular architecture, interfaces, and standards—has helped MSF maintain supply chain responsiveness. Specifically, it (1) enabled time‐consuming, nonessential tasks to be skipped, (2) relieved internal and external bottlenecks, and (3) facilitated better allocation and prioritization. Our analyses also put forward eight moderators, structured in three dimensions (visibility, alignment, and resource orchestration), which can affect the impact of process modularity on supply chain responsiveness. We extend the literature on supply chain responsiveness and process modularity by presenting extensive empirical results suggesting *that* process modularity improves responsiveness in crisis situations, *how* it does so, and *what* moderates this impact. Our study thereby highlights the potential of this strategy and provides operationally relevant insights that could help organizations to implement or to review and redesign their process modularity.

## 1 INTRODUCTION

The world is currently responding to what has been described by the United Nations Secretary‐General António Guterres as “the greatest test since the Second World War” (United Nations, 2020). The unprecedented scale of the Covid‐19 pandemic is presenting a challenge for health product supply chains around the world. Regulatory changes, export bans, border controls, international flight restrictions, and closure of production lines have caused disruptions, leaving western countries short of personal protective equipment (PPE), respirators, medicines, and hand sanitizer. Meanwhile, countries that were already in need of humanitarian assistance before the pandemic are firefighting on multiple frontlines to ensure access to healthcare.

To cope, international humanitarian organizations (IHOs) play a critical role in helping many countries (UNICEF, 2020), but delivering humanitarian aid is challenging. As with many other emergencies,^1^ it requires IHOs to not only scale up their activities significantly to provide additional aid but also to ensure that this does not disrupt their existing development programs (Besiou et al., 2011). Such a balancing act requires supply chain responsiveness (henceforth, responsiveness)—the ability of a supply chain to satisfy the needs of beneficiaries (cf. M. Kim et al., 2013, p. 5602)—with regard to the items needed for relief and development. However, where IHOs are already managing hugely complex supply chains in “normal” times, additional disruptions to demand and supply put them under even greater pressure during emergencies such as the Covid‐19 pandemic.

Many strategies already exist to help maintain responsiveness during disruptions (cf. Sodhi & Tang, 2012). Studies have highlighted the importance of enhancing the modularity of supply chains, products (e.g., Balcik & Beamon, 2008; Kovács & Spens, 2011; Merminod et al., 2014), and services (e.g., Oloruntuba & Gray, 2006). Our study focuses on process modularity, which is achieved when process components or subprocesses “can be reconfigured with little loss of function” (Schilling & Steensma, 2001). This can facilitate swift reconfiguration of processes that may work well in normal times but become too slow, require too many resources, or are too inflexible during disruptions.

Although process modularity has indeed been argued to improve responsiveness (cf. Kleindorfer & Saad, 2005), empirical evidence supporting this is scant, particularly for crisis situations.^2^ Literature has predominantly studied process modularity in manufacturing settings when it is *combined with product modularity*; not when deployed in isolation. One could also *question* whether process modularity is likely to have a positive impact on responsiveness, as reconfiguring processes is by no means easy and may create more problems than it solves, especially when this happens under time pressure. For example, in the context of product modularity, “overmodularization” is a well‐known phenomenon, where “the speed and efficiency gains from modularization will be offset by the increased time spent in the testing and integration phase (…)” (Ethiraj & Levinthal, 2004). In the context of product and organizational modularity, Colfer and Baldwin (2016) similarly stress that “premature modularization misses latent interdependencies, which (…) may cause the system to fail entirely.” One would expect similar risks and trade‐offs with process modularity. As such, it is not only unclear whether process modularity actually “works” in crisis situations but also *what is needed* to make it work—the factors that moderate the relationship between process modularity and responsiveness.

Our exploratory research of the Covid‐19 response of one of the most well‐known medical IHOs—Doctors without Borders (Médecins Sans Frontières; MSF)—allows us to address this gap. We combine qualitative content analysis (QCA), descriptive statistics, and a policy Delphi study to understand how MSF maintained the responsiveness of its health product supply chain. Our analyses (see Sections 3 and 4) show that its supply chain processes can be conceptualized as modular ones, facilitating rapid process reconfiguration in crisis situations. Reconfigurations of this kind are referred to by the organization itself as “stop and switch” or “switching from regular mode to emergency mode.” Interviews with 43 of MSF's staff suggest that such reconfigurations were instrumental for maintaining responsiveness to ensure the continuity of care, the organization's main goal for the Covid‐19 response, a situation that was not the usual short‐term localized emergency for MSF.^3^ This paper sets out to reveal the underlying mechanisms by addressing two research questions: (RQ1) *How does process modularity support supply chain responsiveness?* and (RQ2) *What are the moderators for the impact of process modularity on supply chain responsiveness?*

The Covid‐19 pandemic provides a unique opportunity to study these questions. It caused major supply and demand disruptions, and it is the first crisis to have affected the entire MSF movement simultaneously: each of its three supply centers, five operational centers (details to follow), 77 countries, and 151 missions. As we show, MSF made full use of process modularity to cope with this crisis, reconfiguring each of its processes across missions, operational centers, and supply centers. This provides rich data to address our research questions.

Our work makes three important contributions to the literature on process modularity and responsiveness. First, our study is one of the first studies to present empirical evidence on the impact of process modularity on responsiveness in general and the first to do so for crisis situations. We show how MSF's supply chain processes have a modular structure, with standard interfaces and outputs, and present extensive empirical results supporting that activating, deactivating, and resequencing of process modules helped absorb the shock of the pandemic. Second, we propose specific mechanisms by which process modularity impacts responsiveness: Namely, it (1) enables time‐consuming, nonessential tasks to be skipped, (2) relieves internal and external bottlenecks, and (3) facilitates better allocation and prioritization. Third, we suggest eight moderators of the relationship between process modularity and responsiveness that have not been considered in the literature, which we categorized in three dimensions: visibility, alignment, and resource orchestration. These contributions are also of immense practical relevance, as we discuss in Section 5. Our study also contributes to the literature on managing concurrent emergency relief and existing development programs (Besiou et al., 2011). With a few notable exceptions (e.g., Besiou et al., 2014; Jahre et al., 2016), this “balancing act” has not been considered in previous research.

The remainder of this paper is structured as follows: Section 2 provides a brief overview of responsiveness and the concept of modularity. Section 3 describes our methodology and case. The findings are presented in Section 4 and discussed in Section 5.

## 2 RELATED LITERATURE

### 2.1 Supply chain responsiveness

Supply chain responsiveness has received increased attention from operations and supply chain management (OSCM) scholars in recent years. Because of the growing risks of external disruptions (e.g., earthquakes and flooding), preventing, detecting, and reacting to such disruptions has become a dedicated area of supply chain management (Kleindorfer & Saad, 2005). Supply chain responsiveness is defined as the “ability of a supply chain to satisfy customers’ needs” (M. Kim et al., 2013, p. 5602) and “denotes the capability of a firm to deploy resources available along the supply chain to identify and react to market changes” (D. Kim & Lee, 2010; p. 964). Responsive supply chains ensure appropriate lead times and adequate service quality and quantity (S. Singh et al., 2015).

Many studies provide evidence of factors that affect responsiveness, including collaboration (e.g., D. Kim & Lee, 2010; Squire et al., 2009), organizational hierarchy (Mihm et al., 2010), leanness, and agility (e.g., Holweg, 2005; Lee, 2002; Qrunfleh & Tarafdar, 2013). R. K. Singh (2015) examines 17 critical factors for responsiveness and demonstrates that top management commitment, strategy development, and resource development are the major drivers, followed by trust, collaboration, and information‐sharing. Agility, lead time reduction, and integrated inventory management are also shown to contribute to responsiveness. Modularity is also commonly claimed to enhance responsiveness (e.g., de Waard et al., 2014; Kleindorfer & Saad, 2005; Qrunfleh & Tarafdar, 2013; Squire et al., 2009;).

### 2.2 The concept of modularity

Modularity as a concept can be traced back to Simon's (1962) concept of “near decomposability,” which states that systems can be decomposed into component subsystems (e.g., molecules) to reduce complexity. It is also linked to Starr's (1965) pioneering concept of “modular production” or “the capacity to design and manufacture parts that can be combined in numerous ways” (Starr, 1965, pp. 131–132, in Gupta & Roth, 2007). Weick (1976) proposed a similar concept, “loose coupling,” which refers to systems in which elements are responsive, but physical or logical separateness is preserved, and he presented its advantages, including localized adaptation. In sum, modularity can be defined as “a special form of design which intentionally creates a high degree of independence or loose coupling between component designs by standardizing component interface specifications” (Sanchez & Mahoney, 1996, p. 65).

Modularity enables rapid system reconfiguration by splitting and substituting modules. This provides a powerful tool for mitigating the risks associated with disruptions (Baldwin & Clark, 1997). Creating a modular system involves designing different versions of its components, with different functionalities or performance levels (Sanchez, 1997). For this, modularity relies on three aspects. The *architecture* (also called functional binding or mapping) defines which modules are part of the system and what their functions are. *Interfaces* describe the interaction between the modules. *Standards* are used for testing conformity to design rules. Modules can evolve autonomously, while their interfaces with other modules remain the same (Baldwin & Clark, 1997 Böttcher & Klingner, 2011; Carlborg & Kindström, 2014; Langlois, 2002; Pil & Cohen, 2006).

Literature on modularity has examined various types of modularity, including product, process and supply chain (Fine, 1998, 2005), service (Frandsen, 2017), and organizational modularity (Eppinger & Browning, 2012; Helfat & Eisenhardt, 2004). One branch of literature discusses the relationship between modularity and organizational structure under the “mirroring hypothesis” view (Cabigiosu & Camuffo, 2012; Elia et al., 2019; Salvador, 2007), and the relationship between product modularity and supply chain structure (Davies & Joglekar, 2013; Ro et al., 2007; Ülkü & Schmidt, 2011). Other studies examine the advantages of modularity, including strategic flexibility (Sanchez & Mahoney, 1996), economies of substitution (Garud & Kumaraswamy, 1995; Worren et al., 2002), mass customization and postponement (Hsuan Mikkola & Skjøtt‐Larsen, 2004), increased opportunities for outsourcing (Hsuan, 2003; Schilling & Steensma, 2001), and competitive advantage (Voss & Hsuan, 2009). Our study builds on this literature but focuses, however, on the impact modularity has on responsiveness. While research has predominantly studied this link in relation to *product* modularity, increasing attention is now being given to *process* modularity.

### 2.3 Process modularity

A process is defined as “a structured, measured set of activities designed to produce a specified output for a particular customer or market” (Davenport, 1993, p. 5). It emphasizes *how* work is done rather than *what* is done. Process modularity is achieved when process components or modules “can be reconfigured with little loss of function” (Schilling & Steensma, 2001). Such reconfiguration can take the form of activation, deactivation, decoupling, or resequencing of process modules (Vickery et al., 2016). This requires a “relatively high degree of formalization and codification” (Worren et al., 2002), which can be achieved through visible design rules—rules that define the architecture, interfaces (i.e., input and output), and standards used for processes. Hence, a process module is a standardized process component that usually has few strong ties with other process modules which enables rapid decoupling and resequencing of processes (Fine et al., 2005). Process modularity differs from business process reengineering (BPR), which focuses on radical redesign of business processes (Hammer & Champy, 1993). With BPR, “current processes are *abandoned*, and new and radical processes are developed” (Love & Gunasekaran, 1997). Process modularity does not *abandon* current processes but involves “plug‐and‐play” process reconfiguration using predesigned modules.

In the OSCM literature, modularity has been investigated empirically from various angles. However, most studies investigate process modularity combined with product modularity (Droge et al., 2012; Gualandris & Kalchschmidt, 2013; Jermsittiparsert et al., 2019; Krikke et al., 2004: Patel & Jayaram, 2014; Ro et al., 2007; Thatte, 2013; Vickery et al., 2016), supply chain modularity (Doran, 2003), or both (Voordijk et al., 2006). For example, Droge et al. (2012) demonstrated that process and product modularity can have a positive impact on delivery performance through supplier and customer integration. Thatte (2013) studied modular products, processes, and teams and found a positive indirect relationship between process modularity and responsiveness. Patel and Jayaram (2014) established that process modularity acts as a moderator of the impact of product modularity on operational performance. We identified no studies revealing how process modularity impacts responsiveness when deployed *in isolation* (i.e., without product modularity). Furthermore, the studies that examine the relationship between modularity and responsiveness largely follow a quantitative, *confirmatory* research approach, while there are few qualitative, *exploratory* studies. Studies of this kind are particularly necessary when examining poorly understood phenomena. Our exploratory research aims to fill these gaps. It also helps to address a number of gaps identified by Jayaram and Vickery (2018) in their editorial for the *International Journal of Production Research* special issue on modularity. In general, they highlighted the limited research on process modularity compared to product modularity and called for more empirical evidence. They identified a particular lack of evidence regarding the standardized component interfaces associated with modular structures, the role they play in loosely coupled organizational structures, and whether they decrease the need for coordination and “managerial authority,” as suggested by the “mirroring hypothesis” (Sanchez & Mahoney, 1996). Finally, they also pointed out the need for additional work on the organizational and/or environmental conditions that lead organizations to adopt a modular strategy. We explore these issues using evidence from MSF's response to the Covid‐19 pandemic.

### 2.4 Process modularity in humanitarian supply chains

Ensuring responsiveness is a key objective for humanitarian supply chains (HSCs), but it is also challenging due to the enormous uncertainty inherent in humanitarian crises (Kovács & Moshtari, 2019). Modularity has been advocated as a promising strategy for coping with them (cf. Jahre et al., 2009), as it enhances adaptability (Tomasini & Van Wassenhove, 2009). However, research on modularity in HSCs at most *discusses* or *posits* its impact on responsiveness but does not provide any empirical evidence. Furthermore, it focuses almost exclusively on products. For instance, Chandes and Paché (2010) discuss “modular survival,” referring to standard survival kits as a basic component of responsive HSCs. Scholten et al. (2010) refer to “modules” as a product design principle that enables postponement and leads to agility, while Kovács and Spens (2011) highlight the lack of product and packaging modularization in HSCs.

The only published work, at the time of this research, that discusses process modularity in the humanitarian context is the study by Jahre and Fabbe‐Costes (2015) of the International Federation of the Red Cross emergency response units (ERUs). The authors use a systematic literature review to propose a conceptual framework that illustrates the relationships between physical (product) and organizational (service, human resources, process) modularity, standards, and responsiveness. They put forward a proposition on the link between standards, modularity, and responsiveness but do not present empirical evidence themselves, nor do they discuss its mechanisms and moderators.

**FIGURE 1:**
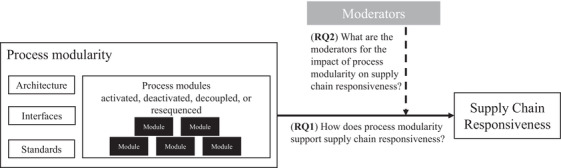
Conceptual framework

Figure 1 summarizes the different concepts discussed in the literature review and the relationships between them. First, the literature emphasizes that modularity requires design rules that define the architecture, interfaces, and standards of processes. Process modules are conceptualized as black boxes that can be activated, deactivated, decoupled, or resequenced. Studies suggest that modularity has a positive impact on responsiveness through factors such as agility, flexibility, and integration. However, there is little evidence on this relationship, its mechanisms (RQ1), and moderators (RQ2). Following Jahre and Fabbe‐Costes's (2015) call for field studies of ongoing operations to strengthen the theoretical foundation underlying this relationship, and also Jayaram and Vickery's (2018) call for more empirical research on process modularity, this research aims to address those gaps.

## 3 METHODOLOGICAL CONSIDERATIONS

### 3.1 Research design

This exploratory study originates from MSF's interest in reviewing its response to the Covid‐19 pandemic and the importance of increasing our theoretical understanding of HSCs. MSF is one of the largest international medical humanitarian organizations, widely recognized for its competence in logistics, and its responsiveness (van der Laan et al., 2009; Vega, 2016). As such, it represents a critical case that is “particularly important in the scheme of things” (Patton, 1990, p. 174) and that permits “*logical generalizations* […] made from the weight of evidence produced” (Patton, 1990, p. 175). Despite MSF's unique structure and position, our findings could potentially be applied to medium and large IHOs involved in both emergency response and ongoing operations, as modular process structures allow for rapid reconfiguration that can be useful when facing emergencies within long‐term crises.

**FIGURE 2:**
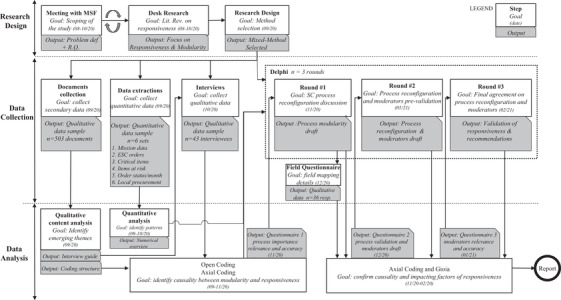
Research design, data collection, and analysis

We follow Sabri et al.’s (2019) collaborative research process to ensure a high level of involvement from practitioners and researchers, better data quality, contextualization, and relevance of the results (see Supporting Information Appendix 7). A team of MSF representatives (henceforth referred to as the MSF Team) was established to codefine the research problem, discuss the research design proposed by the researchers, and plan the data collection jointly. The MSF Team was also briefed on progress throughout the project and presented with preliminary results. The research team has extensive experience of working with MSF in various roles, which provided a thorough understanding of the organization and the potential data sources. This advantage allowed us to adopt a mixed‐methods approach, combining quantitative data (e.g., statistical trends) with qualitative data (e.g., stories and personal experiences), which provides a better understanding of the phenomenon (Creswell, 2015). The quantitative data routinely captured by MSF allowed us, to some extent, to undertake the quantification needed to answer our research questions. This was complemented by relevant documents identified jointly by the two teams and extensive qualitative data gathering and analysis (see Figure 2). Our research can thus be categorized as *QUAL+quant* (Johnson et al., 2007). This approach has been used successfully in humanitarian logistics research that follows a collaborative process with the humanitarian organization being studied (see, e.g., Baharmand et al., 2019, 2020; Charles et al., 2016; Laguna‐Salvadó et al., 2019).

### 3.2 Case description: Organization and context

With nearly 50 years of field experience, MSF is one of the largest and widely recognized IHOs, providing medical assistance to people affected by conflict, epidemics, disasters, or exclusion from healthcare, in more than 77 countries. MSF's medical activities include, for example, surgical interventions, treatment for several diseases, feeding programs for severe malnutrition, and distribution of relief items.

**FIGURE 3:**
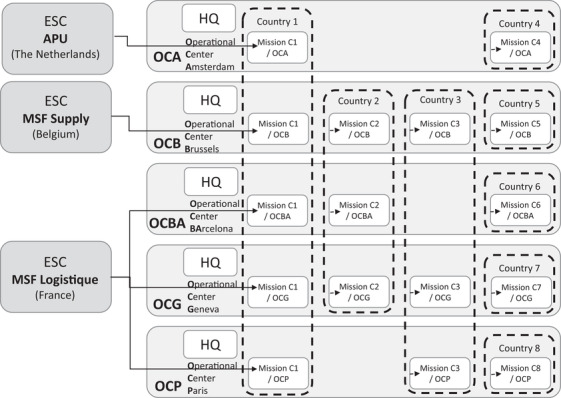
Overview of the MSF organization (visual cocreated by the authors and MSF staff)

As a movement, MSF is organized around five independent operational centers (OCs) or “sections” that directly manage the organization's humanitarian action in the field and decide when, where, and what medical care is needed (see Figure 3). Historically, these OCs were created one after the other to respond to the growing needs of the humanitarian sector. These independent, semiautonomous structures were kept to ensure flexibility and coverage through different legal statuses (e.g., in case of armed conflict different OCs can stand on different sides of the front line; or if one OC is not allowed to work in a certain country anymore, another can take over). The five OCs share a commitment to the MSF principles; they all have the same charters, medical protocols, and items catalog, and work to the same standards in terms of quality, accountability, transparency, item codification, and logistics. However, each OC operates with its own internal structure, management, guidelines, and processes. Joint initiatives, projects, and tools developed across OCs are called *intersectional*. All activities within a country that are managed by one OC are called a *mission*. Multiples OCs could have missions within the same country, and these could be either different activities in the same location (e.g., surgery carried out by Operational Center Amsterdam [OCA] and vaccination campaign by Operational Center Geneva [OCG]) or similar activities in different regions (e.g., the Ebola response across the Democratic Republic of Congo).

Every mission sources medical items from European Supply Centers (ESCs), MSF's highly specialized centers of expertise that ensure compliance with pharmaceutical standards and with national and international regulations and proper packaging and transport of sensitive items. The three ESCs (MSF Supply, MSF Logistique, and the Amsterdam Procurement Unit (APU) are independent entities with their own management, strategy, and operational budget but with similar processes and interfaces; they function as suppliers within the MSF movement. MSF uses this international procurement strategy to ensure that items supplied meet all approved pharmaceutical standards and to eliminate any risk of counterfeit goods or fraud. Overseeing this global structure, the International Office (IO) has an advisory and facilitating role for intersectional cooperation, and the logistics and supply directors of all five OCs and three ESCs come together at the Executive Supply Chain Committee (ESCC). The objectives of the ESCC are to exchange best practices, mutualize resources, take strategic decisions, and coordinate intersectional projects such as information systems and ERP development. Approximately 80% of MSF missions are “regular” programs, long‐lasting missions with long‐term activities carried out over up to 30 years. Regular missions have well‐established supply chains, including a warehouse with entreprise resource planning (ERP) systems, contracts with local transport companies, customs agreements, and a memorandum of understanding with the government. The other 20% are emergency missions, lasting 3 months to 1 year, in response to a sudden need in a location where there are no regular missions or where regular missions cannot cope with the sudden increase in workload.

### 3.3 Data collection

Data sources included semistructured interviews, documentation (e.g., policy guidelines, reports, minutes, emails, etc.), data from the ESCs, a survey, and the outcome of a three‐round policy Delphi study (i.e., written, open‐ended questions, poll results, and discussions). A total of 503 documents and six datasets from five OCs and the ESCC were compiled and shared on a secure virtual drive (see Table 1). We extracted relevant material from the Business Intelligence tool PowerBI and from the internal SharePoint pages. The documents included weekly reports, internal updates, minutes, and briefing notes from each OC and ESC, as well as IO platforms such as MSF's epidemiology research center (Epicenter), Logistics Directorship meetings, the ESCC, the Supply Chain Exchange Interface (SEXI), the MSF Strategic Procurement Platform (MSPP), and the Covid‐19 Supply Taskforce.

**TABLE 1:** Number of documents by month of creation and source

		2019	2020								
MSF entity		All year	January	February	March	April	May	June	July	August	September
IO	Intersectional platforms^a^	12		4	37	50	26	18	5	5	
ESC	APU				1	1					
	MSF Logistique				18	24	17	17	7	5	1
	MSF Supply				5	12	10	7	5	1	
OC	OCA			2	12	10	1	1		2	2
	OCB	8	3		1	5					
	OCBA				1	2					
	OCG	2		2	21	41	22	5	1		
	OCP	32			3	14	10	8	3	1	

Abbreviations: APU, Amsterdam Procurement Unit; ESC, European Supply Center; ESSC, Executive Supply Chain Committee; IO, International Office; MSF, Médecins Sans Frontières; MSPP, MSF Strategic Procurement Platform; OC, operational center; OCA, Operational Center Amsterdam; OCB, Operational Center Brussels; OCBA, Operational Center Barcelona; OCG, Operational Center Geneva; OCP, Operational Center Paris; SCM, supply chain management; SEXI, Supply Chain Exchange Interface.

^a^ (ESCC, SEXI, MSPP, DirLog, ExCom), Covid‐19 Information Hub, Covid‐19 SCM.

A list of interviewees was also drafted in collaboration with the MSF Team, using a combination of nonprobabilistic sampling techniques (i.e., theoretical, purposive, and snowballing). Forty‐three semistructured interviews were conducted online in October 2020 with staff from all ESCs and OCs, working both in the field and at headquarters (HQ; Table 2). The interview guide was prepared by two researchers and thereafter validated by three MSF members in senior supply chain positions. We subsequently performed a pilot interview with an MSF manager to check for clarity and relevance of the questions. The interview guide was finalized afterwards. The interviews (25 in French and 18 in English) were conducted by the principal investigator and lasted 65 minutes on average. The interviews (Supporting Information Appendix 2) focused on the timeline, processes, adaptation, and learning from the organization's Covid‐19 response, and all were recorded, transcribed, and then analyzed using NVivo qualitative data analysis software.

**TABLE 2:** Overview of interviewees

			Specialty of interviewees										
MSF entity			Warehouse	Operation	Supply chain management	Procurement	Purchase	Quality assurance	Transport	Management	Information systems	Pharmacy	Interviewee
IO	IO					**X**							R38, R40
ESC	APU									**X**			R26
	MSF Logistique		**X**	**X**		**X**	**X**	**X**	**X**	**X**			R11, R18, R21, R24, R25, R27, R30, R31, R33, R36, R37, R39
	MSF Supply					**X**	**X**	**X**	**X**	**X**			R17, R19, R32, R42
OC	OCA	HQ		**X**	**X**								R03, R09, R20
		Field			**X**								R12
	OCB	HQ		**X**	**X**						**X**		R14, R15, R29, R34, R35, R41
	OCBA	HQ			**X**								R41
	OCG	HQ		**X**	**X**								R00, R01, R06, R22
		Field			**X**								R05, R07, R10
	OCP	HQ		**X**	**X**	**X**						**X**	R02, R04, R16, R23
		Field			**X**								R08, R12, R28

Abbreviations: APU, Amsterdam Procurement Unit; ESC, European Supply Center; HQ, headquarters; IO, International Office; MSF, Médecins Sans Frontières; OC, operational center; OCA, Operational Center Amsterdam; OCB, Operational Center Brussels; OCBA, Operational Center Barcelona; OCG, Operational Center Geneva; OCP, Operational Center Paris.

The quantitative datasets were extracted from MSF's ERP systems Nodhos and UniField, and described (1) the mission characteristics, (2) information on mission orders (*n* = 13,032) submitted to an ESC (January 2019–August 2020), (3) critical items for the Covid‐19 response (*n* = 63), (4) number of items at risk of shortage for 26 missions (February–June 2020), (5) monthly order status updates on orders for Covid‐19 items (*n* = 881; February–August 2020), and (6) monthly local procurement per mission (€) for January–August 2020 and average monthly local procurement per mission (€) in 2019. We used the datasets to quantify (1) impacts of Covid‐19 on continuity of care, (2) how the number of orders submitted to ESCs and the different components of lead times evolved (pre‐Covid‐19 vs. during Covid‐19), (3) bottlenecks in the supply of Covid‐19 items through the ESCs, and (4) that MSF moved towards local procurement during Covid‐19. Because of the pandemic's emergency status, the quality of the data regarding the distribution process was insufficient and thus this process was removed from the study.

Finally, the principal investigator facilitated a three‐round policy Delphi study (Supporting Information Appendices 4–6) between November 2020 and February 2021 with a Delphi panel constituted of all the ESCC members and additional key representatives within each OC and ESC. This method was chosen as it is known to be a good approach for “correlating views and information pertaining to a specific […] area and for allowing the respondents representing such views and information the opportunity to react to and assess differing viewpoints” (Turoff, 1970, p. 83). Prior to round one, all supply chain processes, that had been reconfigured during the first wave of the pandemic in order to respond to the increasing disruptions and subsequent need for timely access to materials, were mapped using the documentation and interviews. These results were presented and discussed in round one, which focused on the process‐mapping review and preliminary analysis of the impact on responsiveness. The collective output formed the basis for drafting a questionnaire used to assess the impact of process reconfiguration. To validate the details of process mapping at the field level, another questionnaire was sent to all MSF field staff associated with supply chain management. This questionnaire, which investigated potential discrepancies between field and HQ perspectives, was developed by the researchers and confirmed for wording by each OC's supply chain director. Thirty‐two field staff from 32 different missions in 23 countries answered. The Delphi panel confirmed that the sample was representative of MSF missions.

In round two, the results from the questionnaires were presented and discussed. The output of this round was an agreement on process reconfigurations that took place in MSF during its Covid‐19 response. Furthermore, preliminary findings regarding the factors that can influence the impact that modularity has on responsiveness (i.e., moderators), identified through interviews and analysis of documents, for example, were presented and discussed. We built on these insights to create a third questionnaire to investigate further moderators between process reconfiguration and responsiveness. In round three, the impact of process reconfiguration on responsiveness and the moderating factors were discussed and confirmed. The findings from this last round were used to formulate a series of recommendations in a report that was shared and reviewed across MSF.

### 3.4 Data analysis

Different techniques were combined in the data analysis. The first step was to analyze the 503 documents to identify emerging patterns and topics with regard to MSF's response to the Covid‐19 pandemic. Content analysis was used for the purpose of “making inferences by objectively and systematically identifying specified characteristics of messages” (Holsti, 1969, p. 14). This technique involves codifying data into predefined categories to derive patterns (Guthrie et al., 2004). In line with previous studies in humanitarian logistics that have used this technique (cf. Kunz, 2019; Vega & Roussat, 2019), we used a word frequency query with stemmed words to identify the most important topics in the documents. Words such as “Covid‐19,” “supply,” “stock” and “response,” which were found among the most frequently used, were further investigated to understand their use in the documents and used to build a preliminary list of predefined categories, and as basis for the interview guide.

The interviews were coded in NVivo using two levels of coding (Ellram, 1996; Miles & Huberman, 1994). The first level (open coding) focused on the events that happened during the emergency and the mechanisms used to cope with it. All interviewees confirmed the focus on process modularity and related it to “stop and switch” or reconfiguration. The second level (axial coding) focused on the processes that were reconfigured to achieve responsiveness. Both coding from interviews and documents were triangulated to ensure validity. For the quantitative part, we used descriptive statistics of key variables, including lead times (separated into order assessment, order preparation, and transportation), local procurement (€) per month, number of items/order lines “in order” (i.e., ordered but not yet received). We deliberately refrained from using the data to econometrically model the link between modularity and responsiveness, as data to quantify responsiveness are lacking (and so are data for most forms of modularity).

Finally, we used the policy Delphi method to study expert perception on factors that have impacted the relationship between modularity and responsiveness (Turoff, 1970). We followed the Gioia method for structuring data (Corley & Gioia, 2004; Gioia & Chittipeddi, 1991; Gioia et al., 2013) and used NVivo as text analysis software to organize these factors. The transcription from each round was coded at two levels. Open coding was used to identify informant‐centric terms. For each factor frequently mentioned as one affecting the impact of modularity on responsiveness, a “free node” was created using a tentative name (e.g., “Access to data,” “Accurate stock level”). Those were later rewritten in a clear sentence as a first‐order concept (e.g., “Timely access to quality data”). We subsequently used axial coding to categorize the first‐order concepts into researcher‐centric concepts. Here, the free nodes were merged under a “parent node” that represented second‐order themes (e.g., “Information Sharing”) obtained through our literature review (cf. R. K. Singh, 2015). These themes were finally aggregated into three dimensions (e.g., “Visibility”); see Figure 8. To limit confirmation bias, first‐order concepts and second‐order themes were paired independently by the researchers and discussed to achieve consensus.

## 4 EMPIRICAL FINDINGS

The findings discussed in this section describe MSF's process architecture in detail (Section 4.1), examine how this helped maintain responsiveness during the Covid‐19 pandemic (Section 4.2), posit factors that moderate the relationship between process modularity and responsiveness (Section 4.3), and briefly discuss the role of *process reengineering* during the pandemic (Section 4.4).

### 4.1 MSF's preexisting modular processes: Architecture, interface, and standards

**FIGURE 4:**
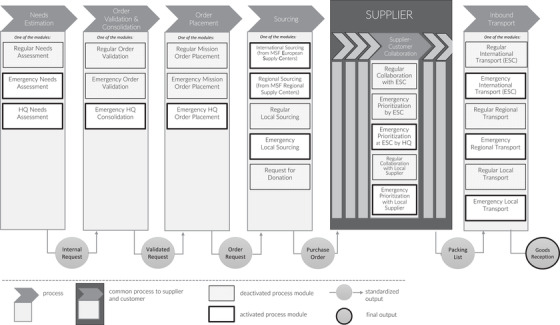
Overview and activation of MSF's modular supply chain process architecture during the Covid‐19 pandemic

Despite slight variations, the supply chain processes for all five OCs can be summarized and conceptualized as displayed in Figure 4, which also shows deactivation and activation of MSF process modules during the Covid‐19 pandemic. This synthesis was based on official OCs’ process documentation and maps. An MSF order goes through a series of six processes, starting with needs estimation and ending with inbound transportation. For each process, two or more process modules are available. These modules can be “mixed and matched” according to the situation or the needs. For example, in the event of an unexpected malaria peak, MSF may deactivate the “regular needs assessment” module and activate the “emergency needs assessment” module. If the standard lead time is too long for the malaria peak response, it can also substitute the “regular international transport” process module with the “emergency international transport” module. Each module has the same input and output and complies with the same standards in terms of quality, transparency, and accountability. MSF's process design can thus be conceptualized as having *an architecture* made up of independent process modules, *standardized inputs/outputs that act as interfaces* between processes, and clear *standards*. The standards are established for each OC and set out in policies and handbooks such as the *MSF Procurement Policy for Medical Products* (2019) or the *Pharmaceutical Support for Local Market Assessment* (2015).

MSF's modular process architecture is the result of *deliberate design efforts* and is supported by multiple projects. Examples include the design of a common ERP tool (Unifield), the implementation of common standards such as the common item codification system (Unidata), the ongoing interoperability project,^4^ and the “process harmonization” project from 2018.

**FIGURE 5:**
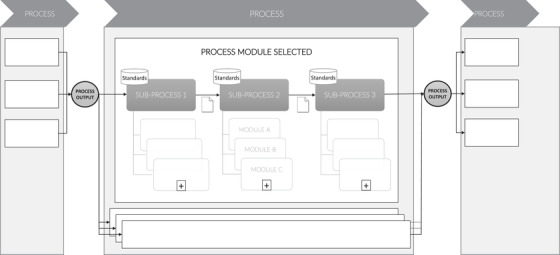
Modular architecture at the MSF supply chain subprocess level

Four other characteristics of MSF's process architecture are worth mentioning. First, modules vary considerably in terms of the *frequency* with which they are used. For instance, the “Emergency prioritization at ESC by HQ” module was created during the 2010 Haiti earthquake and reused for the first time during the Covid‐19 pandemic. Second, modules for the same process can sometimes be run *in parallel*. For example, during a cholera outbreak, MSF supplied cholera‐related items using emergency modules but still used regular modules to supply other items. Moreover, some process modules can be activated *per mission*. For example, emergency modules may be activated by missions with ongoing emergencies while other missions continue to use regular processes. Similarly, for most missions, almost all process modules are activated *at some point or another*. For example, long‐term regular missions like tuberculosis treatment in Mozambique, use emergency process modules over time, as it was the case of cyclone Idai in 2019. Third, as we show in Section 4.4, the architecture is *highly dynamic*: Modules can be added, removed, merged, or updated to allow continuous improvement. Last, the process modularity exists *in all parts of the supply chain* and across the organization—that is, at the field, ESC, OC, and HQ level. For example, like processes, subprocesses can also be modular (see Figure 5).

### 4.2 Relationship between process modularity and responsiveness

Even though MSF is an experienced medical emergency IHO, the Covid‐19 pandemic was unique. First, no medical protocols existed for this new disease, and the information needed to establish them was limited and changed from week to week: “There was no protocol, no expertise with the disease, no stock… We were far from ready” (R22). Second, the scale of the emergency was unprecedented. Never had all existing regular missions been hit by an emergency simultaneously, and all the missions required the same critical items: “Organizationally, we were not prepared to scale up in every country. (…) You can't do that if it's everywhere at the same time” (R09). Third, for the first time all the ESCs and OC HQs were affected by restrictions such as lockdowns, teleworking, and limited human capacity in warehouses: “It was a new situation, and there was much ambiguity” (R16). Finally, this was the first time that the European part of MSF's supply chain had been significantly affected. Market pressures increased on critical items, international transport was drastically reduced, and new export regulations came into place: “It was the first time that something of this magnitude had existed (…) we began to fear that we could not meet all the needs” (R16).

MSF quickly realized that significant effort was needed to ensure continuity of care according to established quality standards. The priority was “to secure supply (…) [for] staff, patients, then all existing projects.” (R22); “It was really to ensure our patients continued to receive the care they need” (R06). The decision was therefore taken to reconfigure MSF's supply chain processes “to speed up to absolutely avoid shortages” (R13). “We had to do everything we could so that no activity stopped” (R02). Official documentation of potential and actual shortages shows that MSF thereby managed to maintain responsiveness: *No single mission* reported interruption of care as a result of shortages. Interviews confirm this: “no activity […] was put on stand‐by [or] was stopped because we did not have the material.” (R05); “we really managed, and actually we managed pretty well” (R01).

The early stages of the interviews and QCA strongly suggested that MSF's ability to reconfigure processes, conceptualized as process modularity, was a critical factor in its success. Indeed, 40 out of the 43 interviewees described and confirmed this. The direct link between process modularity and responsiveness was confirmed by 26 out of 43 interviewees. The following subsections describe this link in detail for the six processes. Table 3 summarizes our findings and the supporting evidence: (1) how each process was reconfigured, (2) which interviewees confirm that this enhanced responsiveness, (3) illustrative quotes, and (4) the authors’ sense‐giving to those quotes. The quotations and the sense‐making were selected by the principal investigator and were crossed‐checked for relevance by the remaining authors, to avoid confirmation bias. We note that, though interviewees rarely use the term “responsiveness” to describe the impact of process reconfigurations, this was the goal set by the organization and shared throughout the movement to respond to the pandemic. Expressions such as “being successful,” “doing good,” “worked well,” “managing,” “ensuring continuity of care,” or “fulfilling the field needs” refer to achieving this goal and can thus be interpreted as indications of responsiveness. Likewise, interviewees do not use the term “modularity” but refer to “stop and switch” (R01) or “activating the emergency mode” (R06, R13, R16, R18, R29, R34, R41) to refer to process reconfiguration through the activation/deactivation of modules.

**TABLE 3:** Summary of findings and supporting evidence of process reconfiguration and its impact on responsiveness, including illustrative quotes and the authors’ sense‐giving to those quotes

**Needs estimation**: Shift from field to HQ level to limit panic‐induced over‐ordering	
Activation of “HQ Needs Assessment”; deactivation of “Regular Needs Assessment” and “Emergency Needs Assessment”	
Confirmed positive direct impact of process modularity (PM) on responsiveness: R00, R06, R14, R16, R35, R40, R41	
The activated process supported the centralized overview of operational needs—a first step toward fair allocation and enabling needs to be met across all missions and not only the first one to place orders.	“We had models to estimate the needs, but we [HQ] needed to check the way they filled in these models. There were missions estimating twenty thousand masks for the first month when their model only said ten thousands. (…) We were in a desperate situation.” (R40)

**Order Validation and Consolidation**: Shift to OC HQ validation to ensure the fair allocation of items in limited supply	
Activation of “Emergency HQ Consolidation”; deactivation of “Regular Order Validation” and “Emergency Order Validation”	
Confirmed positive direct impact of PM on responsiveness: R00, R02, R06, R16, R29, R37, R41	
The activated process was considered as the only way of allowing an overview and fair distribution of the ESC's available inventory. It directly reduced the number of problematic orders (over‐ordering).	“This kind of committee between Supply and Med […] we couldn't have done otherwise” (R02); ”a second or even third check of all these out‐of‐stock; and it's a good thing that they did, because they noticed that some missions were making mistakes in their orders. Even if it was very heavy for the operations department, it was necessary" (R37)

**Order Placement**: Shift order placement process to ensure fair allocation and prioritization of items to avoid stock‐outs across all missions	
Activation of “Emergency HQ Order Placement”; deactivation of “Regular Mission Order Placement” and “Emergency Mission Order Placement”	
Confirmed positive direct impact of PM on responsiveness: R00, R06, R07, R29, R35, R41	
The activated process balanced validated needs and available ESC inventory to prioritize the most urgent orders, resulting in no stock‐outs. The shift removed validation steps and reduced process lead time.	“For the critical items, we arbitrated based on the information we had. Let's say we receive 100 and OCB went through the criticality to supply … 24 for destination X, 10 for destination Y, and so on; in that sense, that's how we never had any issues of capacity or prioritization” (R29)

**Local sourcing**: Activate local sourcing (usually not allowed across MSF missions) to avoid shortages for certain critical items	
Activation of “Emergency Local Sourcing,” “Local Quality Validation,” and “Emergency Local Procurement Financial Validation”; resequencing of “Quality validation” and “Order Request Emission” (quality validation after purchase, but before use); no modules were deactivated	
Confirmed positive direct impact of PM on responsiveness: R00, R01, R03, R05, R07, R08, R12, R13, R14, R16, R20, R28, R29, R34, R35, R41, OCA Covid‐19 Analysis 08 2020, LogIN 11 2020	
In April 2020, all OCs authorized local procurement for some critical items, issuing exceptional local quality validation guidelines, with a simplified local financial validation procedure. Considered a success for responsiveness, it helped avoiding critical shortages and ensured sufficient materials availability (in field) with the best possible quality validation.	“MSF Logistique or MSF Supply, they were not able to supply us here (R05).”We (HQ) authorized to purchase locally, put in quarantine and then validate quality [before use]. It allowed securing inventory, so that we were able to answer with quality items. (R16) “It was fast [and] facilitated for us to search for the provider, to get the validation, and to get the purchases […] It was a good move from MSF.” (R07) ”I think we succeeded a lot because of the local purchase and the few international cargoes that arrived" (R03)

**Regional Sourcing**: Regional sourcing for missions that were previously not part of the regional supply centers [RSCs] portfolio	
Activation of “Regional Sourcing (to MSF Supply Centers)”; the module remained active for missions that had it activated before the pandemic.	
Confirmed positive direct impact of PM on responsiveness: R03, R05, R07, R08, R10,R12, R18, R20, R21, R30, LogIn 11 2020	
With international cargoes pending, continuity of activity was supported through regional sourcing (the RSCs’ extended their portfolio), which was considered a success factor in ensuring items’ availability.	“We [RSC] started to receive orders from many other missions […] ”countries like Yemen [and] Nigeria is way beyond our scope, but we supplied PPE to them […] the missions got what they needed" (R12). “We are not used to doing export import between Mexico and Honduras. But listen, this worked very well" (R05). “Most missions were able to receive their initial PPEs needs on time. (LogIn 11 2020)

**Supplier‐Customer Collaboration**: Shift order prioritization from ESC to OC to make better use of limited resources	
Decoupling of “Order Prioritization Process”; activation of “Emergency Prioritization at ESC by OC HQ”; deactivation of “Regular Collaboration with the ESC,” “Emergency Prioritization with the ESC”	
Confirmed positive direct impact of PM on responsiveness: R00, R06, R11, R16, R21, R29, R37, R41	
OCs (with a better overview of urgency at field level) took over order prioritization and decided (weekly) which order to send to which supplier/ESC for distribution. This improved use of limited resources ensured no missions faced immediate stock‐outs.	“So, the way we stopped the order planification [planning at ESC] and handed it over to the department over there [OCs], it was not bad (…) I think we couldn't have done otherwise. We couldn't have carried on with the planification considering everything that was added, it was not possible.” (R11)

**Field Inbound Transport**: Shift to emergency transport in response to the drastic reduction of available cargo flights	
Activation of “Emergency International Transport (ESC)” and “Emergency partnering with other humanitarian transport solutions” subprocess; deactivation of “Regular International Transport (ESC)”	
Confirmed positive direct impact of PM on responsiveness: R01, R11, R21, R30, R31, R39, R42	
The activated processes enabled the use of ECHO and WFP flights to locations where commercial freight was unavailable. It provided the freight departments with sufficient available transport options to ship all orders in a timely manner (“not being a bottleneck”).	“We managed to align with the ECHO flights, and it was not easy […] because we had the information late, very fast, and at the end of the process. (…) in the end I think we did a great job at the freight department." (R11) “We succeeded using commercial companies or the UN[…]and the collaboration with the UN went really well” (R39) “They [WFP] made full planes for us to the Central African Republic. In Afghanistan, they did five full planes, just for us. That's what allowed it to not be a big bottleneck” (R42).

**ESCs Processes**: Shift to the MSF Strategic Procurement Program (MSPP) to avoid losing purchasing opportunities because of lengthy processes	
Activation of “Purchasing Inter‐ESC Emergency,” “Purchasing emergency,” “Procurement Inter‐ESC,” and “Procurement Emergency”; re‐sequencing of subprocesses from these four modules to shape the temporary module “Taskforce Covid‐19”; no modules were deactivated	
Confirmed positive direct impact of PM on responsiveness: R00, R01, R02, R06, R09, R11, R14, R16, R17, R19, R20, R21, R22, R23, R26, R29 R31, R33, R34, R35, R36, R38, R40, R41, 202008_Lessons learned (*) document “20201106_ESCC”	
The activated and resequenced modules enabled new suppliers’ identification for critical items. 60% of the respondents were positive about the use of the Task Force (*) with a combined purchaser and medical quality assurer team, which improved the sourcing and the use of resources to ensure fast delivery and availability of goods (avoiding critical stock‐outs).	“What changed is the point of entry for the three supply centers in the ESCs” […]" (R17) “Normally, it's a supply [department] issue. But with the taskforce, it was more structured, a bit more of demand planning with quality focus. It really helped, and it fostered collaboration that we never had before between supply and med ops [medical and operations6” (R14). “Without this, we wouldn't have had the level of results that we had” (R19). “Personally, I am convinced that if we had done otherwise, we wouldn't have managed. (…) it was efficient, very efficient” (R31).

Abbreviations: ECHO, European Community Humanitarian Office; ESC, European Supply Center; IO, International Office; MSF, Médecins Sans Frontières; OC, operational center; OCB, Operational Center Brussels; PM, process modularity; RSC, regional supply centers; WFP, World Food Program.

#### 4.2.1 The needs estimation process

The Covid‐19 pandemic posed two challenges for needs estimation. First, “The needs order validation was slowing down the process” (R16). Needs estimation is usually a lengthy process with multiple cross‐checks to avoid over‐ or under‐ordering, while the crisis situation demanded speed. In response, MSF replaced the “Regular Needs Assessment” module with the “Emergency Needs Assessment” module, which removed several checks. Second, the sense of scarcity inflated the missions’ estimation of their needs, as “despite [HQ] communicating the protocol … [the field] didn't calculate as they should have: they ordered more to feel reassured” (R16). MSF therefore activated the “HQ Needs Assessment” module, in which, after consultation with the field teams, HQ staff conduct the assessment of needs and rationalize the estimates of needs accordingly. The activated process modules comply with the needs estimation process standards, the medical protocols, and the organization's transparency requirements. Following the standards, the format of the process output, also remained the same, allowing for smooth interoperability with the subsequent process.

The quantitative data indeed suggest that activation of these modules had an important impact on the organization's lead times and hence its responsiveness. Compared to the average number per month in 2019, the number of order lines formulated, validated, and placed with the ESCs increased significantly at the start of the pandemic: It rose by more than 82% in March) and by 152% in April for high‐priority order lines, and by 27% (March) and 17% (April) for normal order lines. Despite that, the time required to complete the needs assessment process decreased from an average of 25.67 days in 2019 to 20.28 days in 2020. Furthermore, the modules facilitated rationalization and allocation of scarce supplies, which reduced the risk of stock‐outs in the field. This was confirmed in our interviews (see Table 3).

#### 4.2.2 The order validation and consolidation, and the order placement processes

The “Regular Order Validation” process involves a series of checks regarding budget, project codes, substitution (in case of stock‐outs, scarcity, or changes in assortment), and the splitting of suborders, among others. Validation of a typical order requires between four and eight MSF staff. To speed up the process, all five OCs activated the “Emergency Order Validation” module, where only two people are needed to validate an order and fewer checks are required. In addition, since demand for many items exceeded supply, prioritization was essential. MSF therefore activated the “Emergency HQ Consolidation” module, which determines the sequence in which orders are handled. These modules are commonly used for emergency situations and comply with transparency, accountability, and output format standards. In the words of one interviewee, “you go from the regular validation table to the emergency validation table. So yes, it speeds up things!” (R08).

Accordingly, the lead time for order validation was only slightly longer (7.1 in 2019 vs. 7.7 days in 2020), despite the significant increase in the number of order lines (36,691 for March–May 2019 vs. 56,745 for March–May 2020). Many of the field survey respondents (46.9%) agreed (strongly) that “The HQ review of (their missions’) needs was conducted in a timely manner.” Interviewees confirmed that “even if it was very heavy for everyone at the [ESC] operations department, it was necessary” (R37) and “worked well” (R41).

After validation, orders are entered into the ordering system. In the “Regular Order Placement” process, the HQ, the head of mission, and the supply manager are all asked for confirmation before the order is placed with the supplier. In the “Emergency Order Placement” module, however, the supply manager confirmation stage is skipped (complying with the organization's output format and standards). MSF deemed both of these processes to take too long during the Covid‐19 pandemic. Thus, it activated the “Emergency HQ Order Placement” module, which only requires confirmation by HQ. Though this module removed decision power from the field, it shortened the process and freed up time to cope with the pandemic. The benefits of this approach were indicated by interviewees (see Table 3) and the questionnaire responses from field supply professionals, 65.7% (21/32) of whom (strongly) agreed that the “order validation process adaptation was helpful for the field.”

#### 4.2.3 The sourcing process

The modular architecture of the sourcing process was the cornerstone of MSF's response to the Covid‐19 pandemic. The default and preferred process module is “International Sourcing from an ESC,” since it guarantees compliance with medical quality assurance standards. It also drives down prices through volume purchase and long‐term supplier relationships. Nevertheless, four alternative sourcing process modules exist. Missions can employ “Regular Local Sourcing” when faced with import restrictions and when sourcing items with a short shelf life. They can also activate a “Request for Donations” when another MSF mission or IHO is coordinating importation into the country or can use “Regional Sourcing,” which allows them to procure items through an MSF regional platform. Finally, “Emergency Local Sourcing” enables local sourcing when other sourcing modules cannot ensure timely delivery.

**FIGURE 6:**
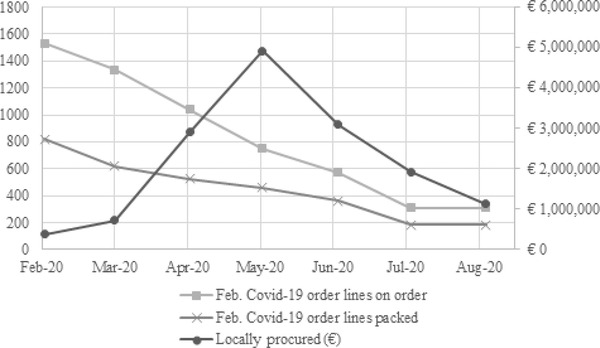
Covid‐19 order lines to ESCs and surge in local procurement

At the beginning of the pandemic, international ESC sourcing could not meet the demand for PPE and several essential medicines. Demand far exceeded the available supply, and international transportation options were limited. As a result, more than 60% of the order lines of items that became critical during the Covid‐19 pandemic and were either “on order” or “packed” in February 2020 still had that status in March 2020, and nearly 40% still had that status by June. This meant that “most missions couldn't receive PPE from the ESC before May, sometimes July” (R20). Therefore, from the end of March, OCs activated “Emergency Local Procurement” for critical items. They “immediately gave instruction to the field that for a couple of months it would be difficult to supply from international supply chains so that they needed to be as self‐sufficient as possible” (R29), which led to a rise in local procurement from March to June 2020.^5^ Figure 6 shows the total number of order lines with Covid‐19 critical items submitted to ESCs whose status was either “on order” or “packed” in February 2020, their status in subsequent months, and the subsequent peak in local procurement.

The switch from international to emergency local sourcing appears to have been critical for maintaining responsiveness. Interviewees confirmed this (see Table 3): “We simply couldn't have continued activities without going for local purchase” (R06); “I think the ease on policy [authorization to activate the process module] on local procurement in every mission was how we made a majority of our mission able to survive during that period […] I think we succeeded a lot because of the local purchase and the few international cargoes that arrived” (R03). It is worth noting that this “ease on policy” involved a change in processes and that the activated process module preexisted and was used before. It was later disactivated as the international purchasing capacity was back to a sufficient level. The majority (84.4%) of the field questionnaire respondents agreed or strongly agreed that “Local procurement was necessary to maintain regular activities.”

#### 4.2.4 The European supply center processes

The Covid‐19 pandemic created three challenges for the ESC process. First, as market tension increased in April, ESC's approved suppliers had limited inventory and many potential clients. ESCs had to act fast to avoid missing procurement opportunities, but the regular process involves lengthy validation and negotiation steps. They therefore activated the “Emergency Purchasing” and the “Inter‐ESC Emergency Purchasing” modules, shortening the negotiation process and the validation lead time for suppliers.

**FIGURE 7:**
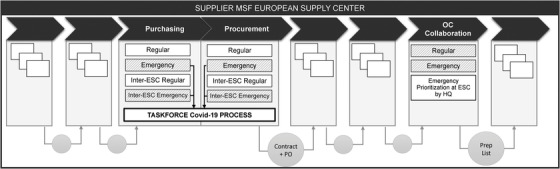
Modularity of selected processes within the ESCs’ supply chain processes during the Covid‐19 pandemic. For a comprehensive mapping of ESC processes, see Supporting Information Appendix 8

Second, the increase in procurement put a significant burden on the quality assurance personnel (QAs) and purchasers. The ESCs therefore activated emergency modules whereby teams of one QA and one purchaser were made responsible for specific items. Flexible resequencing of purchasing and procurement subprocesses was thus authorized, which significantly sped up the process. This module became known as the “Taskforce Covid‐19” module (Figure 7). MSF also decoupled the supplier validation subprocess from this module and allocated it to other staff, as had been done for other emergencies. The organization thereby relieved pressure on a (potential) bottleneck resource: QA personnel and purchasers.

Third, the ESCs’ order‐picking and preparation capacity were also at risk of becoming a bottleneck, which made prioritization essential. Since the OC HQs had reasonable visibility of orders and field priorities (see Section 4.2.2), they stopped the “Regular Collaboration” and “Emergency Prioritization” modules and activated the “Emergency Prioritization at ESC by HQ” module. In this module, ESCs allocate order preparation capacity to OCs, which then select the most critical orders to be prepared. This meant that the ESCs would inform the OCs, indicating, for example, that “we have 1500 lines available for preparation this week. You each have 500 order lines” (R11).

The Delphi participants agreed that “the taskforce was the best way we could have managed despite the lengthy quality validation process” (Delphi 1), and that they “could move on fast, quick and early at the ESC level” (Delphi 2). Interviewees supported this, saying “this QA purchasing pair worked well” (R40) and “If we had done otherwise, we wouldn't have managed. (…) I think it was efficient. Very efficient.” (R31). In all, MSF managed to prevent the order confirmation and preparation steps from becoming significantly longer, despite the crisis: The time required per order went from 40.2 days in 2019 to 40.7 days in 2020 for MSF Supply, and from 45.2 days in 2019 to 50.0 days in 2020 for MSF Logistique.^6^

#### 4.2.5 The international transportation process

From mid‐March to July 2020, the number of options for international shipment reduced drastically. International transport regulations changed possible routes, and the number of flights decreased worldwide. To cope with such constraints, MSF's transport teams stopped the regular modules (international, regional, and local) and activated the emergency modules to ensure timely delivery of orders. The regular process involves first determining the delivery mode and then exploring options with contracted transportation providers. Conversely, the emergency process explores any possible transportation option, relaxing the constraints imposed by the delivery mode selection and transportation contracts. This allowed MSF to bid for World Food Program (WFP) or European Community Humanitarian Office (ECHO) cargo flights, for example. The use of WFP/ECHO flight is a practice used in the humanitarian context, but it is chosen to be a last‐resort process module within MSF. Despite the increased complexity of worldwide transportation, the lead times for transportation remained at almost normal levels during the crisis. On average, it increased by only 1.1 days (29.6 days in 2019 vs. 30.6 days in 2020), which is remarkable given the multitude of constraints. As summarized by interviewees: “in the end I think we did a really great job at the freight department” (R11) and “we succeeded using commercial companies or the UN” (R39).

### 4.3 Factors that moderate the influence of process modularity on responsiveness

**FIGURE 8:**
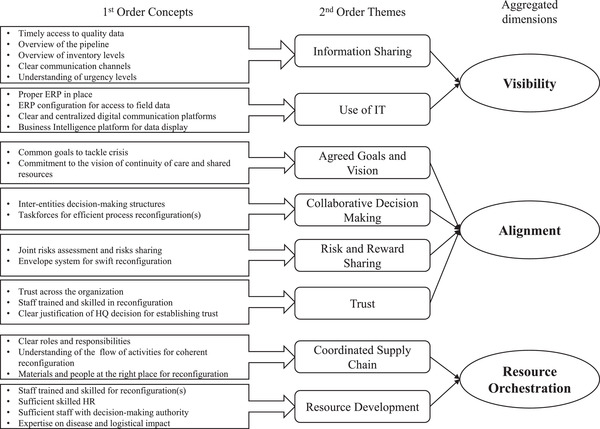
Factors that seem to moderate the influence of process modularity on responsiveness (based on Corley & Gioia, 2004; Gioia et al., 2013)

As argued, reaping the benefits of process modularity is not trivial. Our data suggest eight factors that affect (i.e., moderate) the extent to which process modularity impacts responsiveness (Figure 8). These factors can *enable* a process reconfiguration, decrease *implementation delay*, and/or *improve execution* of reconfigured processes. By applying the Gioia method, we were able to identify and group these moderating factors and related insights during our data analysis (yielding informant‐centric terms) and thereafter conceptualize them during the Delphi rounds (yielding researcher‐centric concepts). The concepts were based on R. K. Singh's (2015) model of responsiveness. We thereby advance understanding of how the “critical factors” put forward in this work impact responsiveness (i.e., they do so, among others, by moderating the influence of process modularity on responsiveness). All findings were validated by the Delphi panel.

#### 4.3.1 Visibility

This dimension concerns the extent to which various actors within MSF's supply chain “have access to or share information which they consider key or useful to their operation” (Barratt & Oke, 2007, p. 1218). The interviews and the Delphi study suggest that *information sharing* enhanced the positive impact of process modularity in several ways. First, information sharing about the level of urgency enables swift decisions to be made about which process modules to activate or deactivate, decouple, or resequence. Second, it ensured that information flows between OCs, ESCs, and fields could be swiftly (re)synchronized with reconfigurations. For example, having access to inventory and pipeline data was crucial for the HQ needs assessment module and the local procurement module (to guide local purchasing decisions). As emphasized by one interviewee, “[One OC] had one person that had access to all the data [and] could respond much faster than the others. It was more efficient” (R21). Third, well‐established information communication channels ensure that messages with instructions or details on the reconfiguration are received faster and are unambiguous. When the communication channels were not clear, reconfiguration took longer and information‐sharing activities that were part of a process module were less efficient, as confirmed by the Delphi panel.

The Delphi panel and interviewees also stressed that the *use of IT* solutions such as SharePoint, PowerBI, and ERP Unifield was essential for accessing and sharing information and for communication across the organization. One respondent stated: “our first bottleneck was inventory visibility, but at least we had Unifield (ERP). Other OCs didn't have Unifield in place, and they struggled much more. We could take decisions and prioritize faster with it” (R06). When such tools were not available, MSF resorted to inefficient “work‐around” solutions (e.g., using Excel spreadsheets and emails).

In short, information sharing and use of IT can impact the implementation time for a process reconfiguration and the speed of the process module itself—that is, they can moderate the impact of process modularity on responsiveness.

#### 4.3.2 Alignment

This dimension concerns the organization's capability to “flexibly adjust its configuration to align the objectives of all members” (Dubey & Gunasekaran, 2016). Alignment is crucial when part of the organization has concerns about participating in a reconfiguration or is disincentivized from doing so, which can block or delay implementation. For example, this could occur when the reconfiguration changes the locus of decision‐making, concentrates it in the hands of a few people, or leads to various kinds of risks (e.g., a cost increase). Our data put forward four moderators that support alignment and thus enhance the chances of reconfigurations being implemented rapidly and speeding up the process modules themselves.

The first is to ensure *agreed goals and vision* across the organization. Despite all OCs being independent, the Delphi panel confirmed that the OCs discussed priorities together, and agreed that continuity of care came first and that additional Covid‐19 activities should come second. The OCs also agreed on the goal of allocating scarce resources equally or according to the level of urgency. This led to swift agreement on process modules that would facilitate allocation and prioritization (e.g., HQ needs assessment, emergency HQ consolidation, and emergency prioritization at ESC by HQ).

The second is to facilitate *collaborative decision‐making* regarding reconfigurations. As explained by one interviewee, “after a week, 10 days, we realized that [common goals] was not going to be enough, but that it was absolutely necessary to sit with the five OCs and the three ESCs” (R31). This led to the formation of an interentity structure (i.e., activation of the ESC Taskforce Covid‐19 module) that was instrumental for swift collective validation and confirmation of emergency module activations. For example, the taskforce supported collaborative decision‐making to swiftly activate the emergency local procurement module upon international sourcing unavailability. Where there was noncollaborative decision making, misunderstandings or bottlenecks occurred, which led to inefficiencies and missed sourcing opportunities (see Section 4.2.4).

Third, mechanisms for *risk and reward sharing* can significantly reduce the barriers to process reconfigurations. For example, the financial risks associated with the inter‐ESC emergency purchasing module were reduced by sharing those risks across entities, which supported its implementation. MSF also reduced the potential risks of reconfigurations through its so‐called “envelope system,” a flexible budget set aside to support the activation of emergency modules at all levels. For example, the envelope system supported the activation of the local procurement module by giving field teams extra, unallocated budget.

Finally*, trust* appears to play a key moderating role. Several process modules change the locus of decision‐making. For example, they moved both needs assessment and order placement from the field to HQ, making OCs (instead of ESCs) responsible for preparing orders, and tasked field staff (instead of ESCs) with validating quality for local sourcing. Decision power thereby shifted to different and/or fewer people, and here trust is essential. For example, while activating emergency local sourcing, some OCs did not activate the “prequality validation” subprocess module (because of mistrust in medical quality‐validation skills at field level), so the HQ quality validation bottleneck was not resolved. This led to long delays in receiving email responses and missed procurement opportunities.

In summary, agreed goals and vision, collaborative decision‐making, sharing of risk and reward, and trust can reduce the barriers and delays associated with process reconfigurations which evidently enhances their impact on responsiveness. That is, they can moderate the impact of process modularity on responsiveness.

#### 4.3.3 Resource orchestration

The third aggregated dimension relates to how organizational resources are orchestrated, that is, how they are managed and coordinated (Christopher, 2005). According to the Delphi panel, *supply chain coordination* was one of the keys to swift implementation of reconfigurations. This involves first ensuring that resources are put “in the right place” to implement reconfigurations and execute the reconfigured processes. It also requires clearly defined roles and responsibilities to ensure timely and efficient decision‐making once modules have been activated. Third, deciding upon reconfigurations requires a sound understanding of how process modules affect one another, for example, to avoid “pushing bottlenecks.” Such examples include activation of the local quality validation module to avoid HQ bottlenecks or the activation of the ESC's emergency prioritization by OCs module.

The feasibility and impact of many process reconfigurations also depend on *resource development*: They may require a significant increase in resources or different deployment of the available resources. For example, activating the HQ needs assessment and order placement modules significantly increased the workload at HQ. Similarly, when the local procurement and quality validation subprocess modules were activated, the workload at the field level changed significantly. Further, an increase in staff with decision‐making authority was needed for several process modules, as showed by the bottlenecks created when activating the local procurement subprocess modules without increasing field managers’ signatory authority. The extent to which such reconfigurations impact responsiveness hence depends on the amount of resource redundancy and flexibility.

### 4.4 The dynamic nature of modular processes

The modular architecture used by MSF is not static. At times processes may need to be updated, adapted, removed, or added. The Covid‐19 pandemic is a good example of this, as the magnitude and unique nature of the emergency required MSF to make slight adaptations to some of the process modules.

Two examples can be found within the “Emergency Local Procurement” module. First, in the original module staff working at HQ or at MSF's regional platform were tasked with validating the quality of all local medical supplies: “Traditionally it had been centralized (…) assuming the risk that some of the supply may not be of the quality that we would normally expect” (R22). Because of the already heavy burden on staff, activating this process would have led to bottlenecks if it had not been modified. Therefore, MSF added a subprocess,“Field Medical Validation,” whereby OCs classified items based on the level of risk to quality and allowed urgent low‐risk items to be validated locally. Second, MSF normally requires medical quality validation to occur before financial validation. Because of market pressures and the volatility of supply availability during the pandemic, this could easily have led to missed procurement opportunities. MSF therefore created a subprocess called “Prequality Financial Validation,” which allowed purchasing to go ahead and items to be quarantined before quality was validated. The organization thereby accepted financial risks to avoid life‐threatening shortages.

These adjustments show that applying BPR to process modules helped ensure MSF maintained its responsiveness during the pandemic. Interestingly, it appears that the organization's modular architecture made it easier to adjust a process (e.g., by adding a new subprocess) and fit it into the overall architecture, because there were already predefined standards and interfaces.

## 5 CONCLUDING DISCUSSION

Processes that work well in normal times can present a huge stumbling block when there are supply chain disruptions. They may be too slow, require too many resources, or be too inflexible to maintain responsiveness. Process modularity is a commonly suggested mitigation strategy: It allows “plug‐and‐play” reconfiguration of processes using predesigned modules. However, doing this under time pressure is by no means easy. Reconfigurations take time and may cause more problems (e.g., confusion and conflict) than they solve, as highlighted by concerns on “over‐modularization” (Ethiraj & Levinthal, 2004) and premature modularization (Colfer & Baldwin, 2016). Empirical evidence supporting the notion that process modularity enhances responsiveness and when it does so (i.e., what the moderators are) is indeed scarce, particularly for crisis situations. Studies have so far almost exclusively provided evidence on the impact of process modularity when combined with product modularity (e.g., Jermsittiparsert et al., 2019; Vickery et al., 2016; Voordijk et al., 2006).

We aim to fill this gap by exploring how process modularity helped MSF maintain responsiveness during the Covid‐19 pandemic. We did this using QCA, supply chain data relating to over 151 missions in 77 countries, 43 interviews with field and HQ staff, and a policy Delphi study. In this section, we summarize and discuss our three main contributions.

First and foremost, we are the first to provide empirical results supporting that process modularity does indeed drive responsiveness during crisis situations. The Covid‐19 pandemic put MSF's supply chain under unprecedented pressure. In response, MSF decided to activate, deactivate, decouple, or resequence many of its predesigned process modules. This, as our data suggest, limited the impact of the disruptions on lead times, enabled prioritization and allocation of scarce resources, and facilitated the temporary move to local contingency procurement. As a result, no single mission experienced interruption of care due to shortages. Given the immense complexity of reconfiguring a process in such a large organization (>45,000 full time equivalent (FTE) in 2019) under time pressure, this is a considerable achievement. We therefore bring to the attention of academics and practitioners a viable strategy for ensuring supply chain responsiveness, one that has surprisingly seldom been explicitly considered or studied.

We are also the first to study *how* process modularity improves responsiveness in crisis situations. The case of MSF suggests three mechanisms. First, process modules can be designed to reduce lead times by *skipping time‐consuming nonessential tasks*—tasks that are important enough to be executed in “normal” times, but which benefits do not justify the lead time increase in “abnormal” times. For example, MSF‐activated modules that reduced the number of quality checks for needs estimation, order validation, and order placement processes. Second, modules can be designed to *relieve potential bottlenecks*, thereby mitigating the risk of serious delays. Such bottlenecks can be internal or external. For example, by moving to local medical validation, MSF reduced the pressure on HQ staff undertaking quality validation, which had become an internal capacity bottleneck. The limited availability of supply and transportation options had created external bottlenecks, and these were relieved by activating inter‐ESC emergency purchasing, local sourcing, and emergency transportation. Furthermore, process modules can be designed to *facilitate the prioritization, allocation, and rationing of scarce resources* in line with needs. For example, process modules activated by MSF enabled the sequence in which orders were handled to be optimized and facilitated rational allocation of inventories to orders and of order preparation capacity to order lines. Similarly, the organization addressed over‐ordering issues by instigating a process in which needs assessment was undertaken at the HQ level.

Third, we not only examine *whether* and *how* modularity impacts responsiveness but also advance understanding of *how one can increase this impact*—that is, the moderators of this relationship. Previous research has studied only one moderator: complexity (of a production process; Vickery et al., 2016). Our data suggest eight additional moderators, grouped in three dimensions. First, *visibility*—for example, in relation to levels of urgency, inventory levels, pipelines, or bottlenecks—helps to ensure rapid decision making on which process modules to activate, deactivate, decouple, or resequence. It also speeds up the reconfiguration itself by ensuring that information flows can be reconfigured quickly in response. Second, *alignment* is crucial to reduce barriers to or delays in reconfiguring processes. This is especially relevant when part of the organization is disincentivized from participating in a reconfiguration—for example, when the reconfiguration changes the locus of decision making, concentrates decision‐making in the hands of a few people, or introduces certain risks (e.g., a cost increase). Trust, shared goals and vision, collaborative decision‐making, and mechanisms for sharing risk and reward help mitigate these issues. Third, process reconfigurations require swift *resource orchestration*. For example, they can require staff to perform more tasks or different tasks and to take on different responsibilities. Developing resource flexibility and redundancy is therefore key, as is taking account of resource availability when deciding *which* process modules to activate, deactivate, decouple, or resequence (to avoid “pushing bottlenecks”). The eight moderators we identified challenge the technical view of process modularity found in the literature (cf. Gualandris & Kalchschmidt, 2013; Vickery et al., 2016) and offer concrete guidance to IHOs striving to reap maximum benefits from modularity: Doing so is not just a matter of optimizing the architecture, interfaces, and standards for process modules. It also requires a review and potentially a redesign of other tangible and intangible resources. We also advance understanding of how “critical factors” (see R. K. Singh, 2015) impact responsiveness.

The relationships, moderators, and mechanisms suggested by our exploratory analyses could help spur much‐needed future confirmatory research on process modularity. Our research is limited in that it focuses on only one IHO. It is important to note that MSF differs from many other IHOs in various ways, including in the length of its missions (93% of the missions studied had already been going on for at least over 1 year when the pandemic started) and the limited amount of earmarked funding. Additionally, the disruption we studied was unprecedented in its complexity. Though the mechanisms that explain the relationship between process modularity and responsiveness may also apply to other organizations and disruptions, the *strength* of the relationship may differ. Various factors may impact this, including the duration of “regular” processes (i.e., the opportunity to gain speed by activating a faster process module), resource availability (i.e., whether there are opportunities to relieve resource bottlenecks by reconfiguring processes), and variability in the urgency of tasks or orders (i.e., the opportunity to improve prioritization of orders and allocation of resources through process reconfigurations). Future research could also assess the validity of our results for different IHOs and different levels of complexity but also for commercial organizations facing similar disruptions.

Our results also beg the question whether process modularity benefits commercial organizations in “normal” (i.e., noncrisis) situations. Literature suggests that improved supply chain responsiveness gives companies a competitive advantage, for example, by being able to swiftly respond to customer requirements and market dynamics (see, e.g., Asamoah et al., 2021). Future work could assess whether and how process modularity impacts responsiveness to attract, satisfy, and retain customers, and thereby increase business performance. Follow‐up research could also use quantitative data and econometric models (e.g., surveys data and structural equation models; cf. Patel & Jayaram, 2014) to validate our findings and assess the relative impact of the moderators. Insights from such studies could help practitioners prioritize which moderators to review in their organization. This avenue also reveals a need for future research examining how to measure process modularity, building upon extensive literature on measurement in the context of product modularity (Gershenson et al., 2004; Salvador, 2007) and modularity valuation (Baldwin & Clark, 2000, 2002). Finally, there is a need for future work describing the *process of developing process modularity*. Insights on this matter would be beneficial for other organizations implementing this strategy.

Our exploratory study supports that process modularity has an impact on responsiveness and suggests both *how* it does so and *what* moderates this impact. We thereby lay the groundwork for future confirmatory research on process modularity, highlight the potential of this strategy, and provide operationally relevant^7^ insights that could help practitioners to implement or to review their process modularity. By design, our paper is the result of an intensive, collaborative research project with MSF, which helped support the organization's goals and ensured that insights are actionable. We extensively discussed our insights with the organization and shared them among others in the form of a report describing for each process what happened, success factors, areas of improvement, and recommendations. This led to several concrete changes or “quick wins” (e.g., a platform for inter‐OC collaboration was revamped to support modular processes) between the first and second waves of the pandemic. It also reaffirmed the importance and the role of process modularity, which MSF has been developing and using for many years, and induced additional investments to improve it. More importantly, our project helped MSF make explicit its tacit knowledge on their “stop and switch” practice, providing the organization with a terminology to communicate about it, and yielding actionable insights and a concrete “toolbox” to reap maximum benefits from it. Our results play a key role in the organization's ongoing 2023–2025 strategy formulation efforts, in which MSF is examining how to better exploit the moderators, and what other delays, bottlenecks, and prioritization/ allocation challenges can be addressed through process modularity. Beyond MSF, other organizations also using modularity (e.g., the Red Cross ERU system; Jahre & Fabbe‐Costes, 2015) are likely to benefit from the experience drawn from the MSF study. Finally, in line with Van Wassenhove (2006), we posit that it is not only other IHOs that can learn from those insights: Businesses clearly also have to deal with high‐impact, low‐probability events—the Covid‐19 pandemic being a prime example—and can learn from humanitarian organizations, for which this is a core competency.

## Supporting information

Supporting Information

## ENDNOTES

1 For example, balancing malaria peaks and ongoing primary health care programs, fighting cholera emergencies while maintaining regular maternal care programs, and providing relief after a tsunami while continuing TB/HIV programs.

2 Only one paper (Jahre & Fabbe‐Costes, 2015) considered process modularity, but it was neither the focus of the study nor was it studied in response to a specific crisis.

3 Under MSF terms, emergencies activate specific resources through preexisting emergency processes to respond to a need. The Covid‐19 pandemic was not defined as only an additional specific need, but the mechanisms applied to switch between regular operations and emergencies were used to ensure the continuity of care under the disruptions caused by the pandemic.

4 The aim of this is to provide various ways of increasing the systems interoperability, internally defined as “the ability to systematically produce a coherent result for end users, not necessarily requiring a full alignment on systems and process.”

5 For reference: In 2019, the average value of local procurements was €1.7 million, a large share of which is spent in December and January. The order increased, mentioned as March–June, is showed by the increase in expenses paid upon goods reception: therefore, reflected on the following month.

6 APU data are excluded because this unit was preparing to move and had limited capacity in 2020.

7 As this research followed the collaborative methodology for humanitarian supply chain research (Sabri et al. 2019), this helped to ensure the operational relevance of the moderators we present.
