# Supplementary material for: Process modularity, supply chain responsiveness, and moderators: The Médecins Sans Frontières response to the Covid‐19 pandemic
Source: Prod Oper Manag. 2022 Mar 3:10.1111/poms.13696. Online ahead of print. doi: 10.1111/poms.13696 (PMC9115391; doi:10.1111/poms.13696)

ONLINE APPENDIX

PROCESS MODULARITY, SUPPLY CHAIN RESPONSIVENESS, AND MODERATORS: THE MEDECINS SANS FRONTIERES RESPONSE TO THE COVID-19 PANDEMIC

APPENDIX 1 : ACRONYM LIST

APPENDIX 2 : INTERVIEW GUIDE

APPENDIX 3 : GIOIA EXTENDED VERSION

APPENDIX 4 : DELPHI QUESTIONNAIRE 1

APPENDIX 5 : DELPHI QUESTIONNAIRE 2

APPENDIX 6 : FIELD QUESTIONNAIRE

APPENDIX 7 : SABRI’S COLLABORATIVE METHODOLOGY

APPENDIX 8 : SUPPLY CHAIN PROCESSES’ MODULAR ARCHITECTURE AT EUROPEAN SUPPLIER CENTERS

APPENDIX 1: ACRONYM LIST

APU – Amsterdam Procurement Unit, European Supply Center in Amsterdam, the Netherlands
ESC – European Supply Center
ESCC – Executive Supply Chain Committee
EPREP – Emergency Preparation
IHO – Internal Humanitarian Organization
MSF – Médecins sans Frontières
MSFL / MSF Log – MSF Logistique, European Supply Center in Bordeaux, France
MSFS – MSF Supply, European Supply Center in Brussels, Belgium
MSPP – MSF Strategic Procurement Platform
OC – Operational Center
OCA – Operational Center Amsterdam
OCB – Operational Center Brussels
OCBA – Operational Center Barcelona
OCG – Operational Center Geneva
OCP – Operational Center Paris
WFP – World Food Program

APPENDIX 2 : INTERVIEW GUIDE

Interview Guide

I. INTERVIEWEE

0. Name, OC/ESCs, country, position
0. Consent to recording
1. Description of job normally
2. Task and job position - Role played during the C19 response
    a. How was it different from normal?
    b. How did it change on-going activities? Were they maintained or were those put on standby?

II. VIEW OF C19 IMPACT PER PROCESS

3. How was needs assessment and planning impacted?
4. How were procurement activities affected? / Affecting you?
5. How did the safety stocks and EPREP stocks “performed”?
6. How were transport affected / affecting you?

III. TIMELINE VIEW

7. Can you recall events from March?
8. April seemed to be the peak; do you agree? What was going on?
9. Can you discuss how May unfolded? Was it still the peak in your opinion?
10. Why activity decreased in June and July?
11. For you, was August back to normal or not?

IV. INTERNATIONAL VIEW

FOR OCs and ESCs STAFF

12. What countries had the response to covid that you judged the most difficult?
13. Which countries did good?
14. Which countries had the most challenge to maintain on-going activities?
15. Which countries manage the balance the best?

FOR FIELD STAFF

16. The response in your country
    a. What made the response easy in your country of activity? government, customs, Ministry of Health, infrastructure etc….
    b. What made the response difficult in your country of activity? government, customs, Ministry of Health, infrastructure etc….
    c. What was the challenge to maintain on-going activities?
    d. How did you manage to balance Covid activities?

V: BEST PRACTICES

17. What would you do again? as individual, entity and movement
18. What would you do differently? as individual, entity and movement

APPENDIX 3: GIOIA EXTENDED VERSION

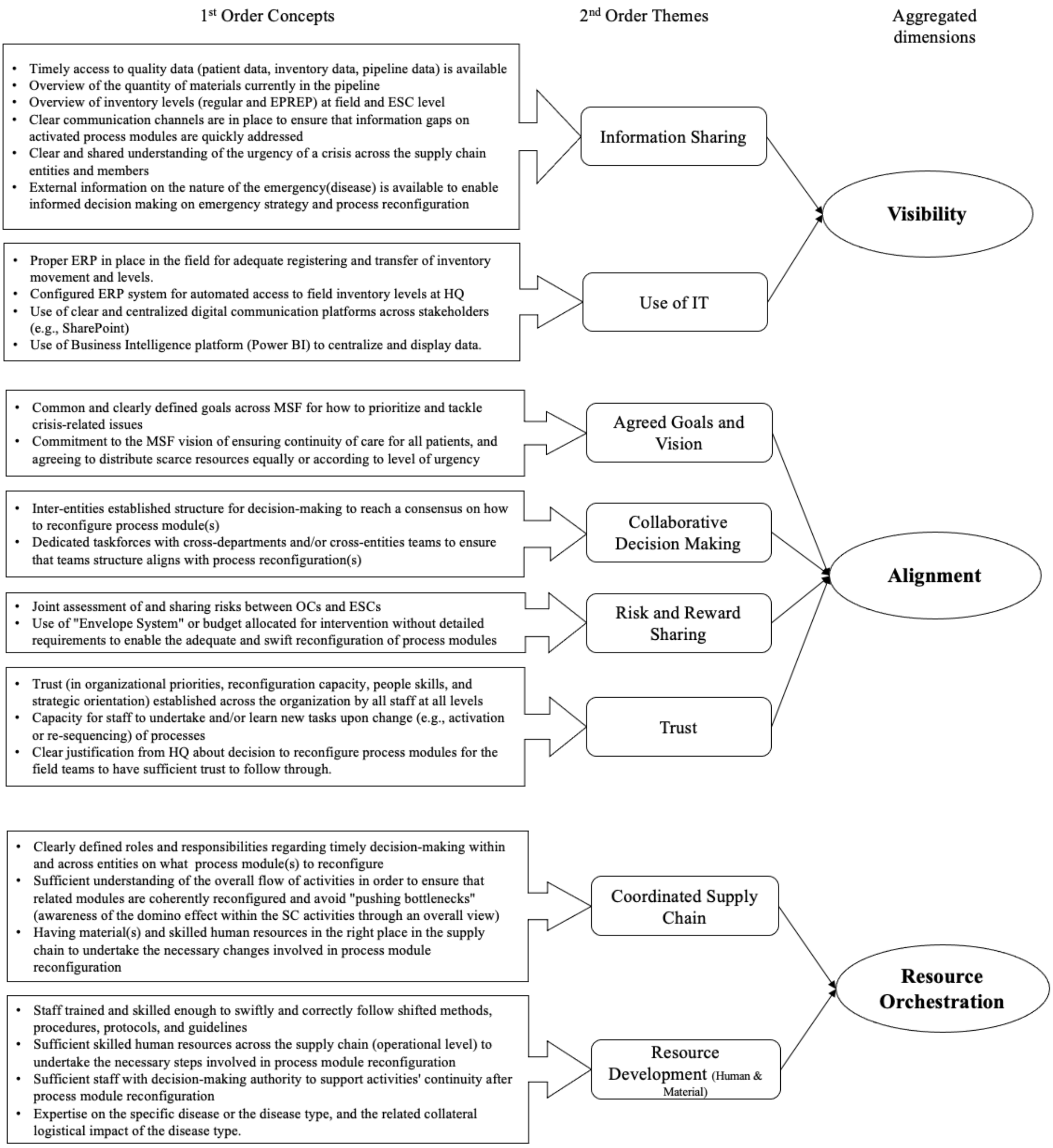

APPENDIX 4: DELPHI QUESTIONNAIRE 1

Dear All,

Thank you for attending the first iteration of the Delphi yesterday.
Please find here under the instructions to fill in the following taps, in oder to prepare the second round and adjust the analysis to your needs.
Thank you in advance for your time,
Best Regards,
The HUMLOG Insitute Team

Instructions

Please use the following process to evaluate each level of the frameworks.
Please evaluate the list of processes that were updated/ changed/ added for the Covid-19 response, and rank them in line of their relevance, importance and accuracy **as part of the lessons learned**

A. Input you perspective on each item relevance, importance and accuracy.
B. Please add your comments, concerns and insights to the comments colums of the process tabs when necessary.
A. Rate which areas of recommendation should be prioritized for operational recomendations by the reseach team.
D. Please add your comments, concerns and insights to the comments colums of the recommendation tabs when necessary.

**Relevance: of the updated/changed/added process to the lessons learned**	
**YES**	- Relevant to the analysis and the lessions learned<br>- Must/Should be included
**NO**	- Insignificantly relevant or No relevance<br>- Should not be included or Could be included with limted added value

**Importance: of the updated/changed/added process to the lessons learned**	
**YES**	- Has highlly significant bearing for the lessons elarned<br>- Contributes substantially to the inputs from the analysis framework<br>- Must be fully explored further
**NO**	- No priority<br>- No measurable effect, has little importance or importance to a relatively on the overall organisation<br>- Not a determining factor to major issue<br>- Should be dropped as an item to consider

**Accuracy: of the wording of the updated/changed/added process to the lessons learned**	
**YES**	- All necessary information is included and underlying concept for the item is easy to understand<br>or Minor information missing but does not substantially affect understanding of underlying item concept<br>- All terms used are widely understood by the majority of the sector, or specific terms are used but does not cause substantial mis-understanding of the item<br>- Wording is in line with rest of framework
**NO**	- Substantial missing information with unclear underlying concept<br>- Item is too easy to confuse with other item(s) with substantial amount of information that is irrelevant to the underlying concept of the item<br>- Unclear terminology not in line with the organization<br>- Requires a re-write

Priority

**HIGH**	The recommendation must be the focus of further detailed recommendations by the reserach team.
**MEDIUM**	The recommendation could be the focus of further detailed recommendations by the reserach team.
**LOW**	The recommendation does not need further detailed recommendations by the research team.
**OUT OF SCOPE**	The recommendation is out of scope and shoudl be removed.

Colonne1	#	Process adapted during C19 pandemic	Actor 1 FIELD	Actor 2 OC HQ	Actor 3 ESC	Actor 4 IO	Intersect° Manageme	Success Factor	Problem Factor	Impact 1 Cost	Impact 2 Time	Impact 3 Quality	RELEVANCE	IMPORTANCE	ACCURACY
NEEDS	100	Needs Assessment and Planning											Yes	Yes	Yes
NEEDS	**110**	**Needs Assessment**											Yes	Yes	Yes
NEEDS	111	Visibility on mission inventory and consumption reporting	•					•		•	•		Yes	Yes	Yes
NEEDS	112	Medical protocol definition		•		•			•	•	•		Yes	Yes	Yes
NEEDS	113	Calulation method guideline defintion		•		•			•				Yes	Yes	Yes
NEEDS	114	Preliminary estimate calculation	•						•				Yes	Yes	Yes
NEEDS	115	Preliminary estimate communication to OC	•						•				Yes	Yes	Yes
NEEDS	**120**	**Forecast establishement**											Yes	Yes	Yes
NEEDS	121	Preliminary estimates verification		•					•		•		Yes	Yes	Yes
NEEDS	122	Revised estimates definition		•					•		•		Yes	Yes	Yes
NEEDS	123	Consolidated forecast decision making and validation		•					•		•		Yes	Yes	Yes
NEEDS	124	Consolidated forecast communication to IO		•					•		•		Yes	Yes	Yes
NEEDS	125	Consolidated forecast decision making and revision				•	•		•		•		Yes	Yes	Yes
NEEDS	125	Consolidated forecast revision to ESCs				•	•		•		•		Yes	Yes	Yes
NEEDS	**130**	**International orders placement**											Yes	Yes	Yes
NEEDS	131	Validated estimates communication to the field		•				•		•	•		Yes	Yes	Yes
NEEDS	132	International orders placement	•	•				•			•		Yes	Yes	Yes
NEEDS	133	International orders review		•				•	•		•		Yes	Yes	Yes
NEEDS	134	International orders decision making and validation		•				•	•		•		Yes	Yes	Yes
NEEDS	**140**	**Prioritization of Orders**											Yes	Yes	Yes
NEEDS	141	Urgency levels definition for pending orders with the field	•	•				•	•		•		Yes	Yes	Yes
NEEDS	142	Consolidation of urgency levels		•				•	•		•		Yes	Yes	Yes
NEEDS	143	Definition of preparation capacity			•			•			•		Yes	Yes	Yes
NEEDS	144	Request for weekly prioritization to Ocs			•			•			•		Yes	Yes	Yes
NEEDS	145	Weekly prioritization of orders definition		•				•			•		Yes	Yes	Yes
NEEDS	146	Weekly prioritization of orders communciation to ESC		•				•			•		Yes	Yes	Yes
NEEDS	**150**	**International pipeline and inventory visibility**											Yes	Yes	Yes
NEEDS	151	Weekly reporting of ESC pipeline and inventory levels			•			•	•	•	•		Yes	Yes	Yes
NEEDS	152	Pipeline and inventory levels communication to IO			•			•	•	•	•		Yes	Yes	Yes
NEEDS	153	Pipeline and inventory levels communication to OC			•			•	•	•	•		Yes	Yes	Yes
NEEDS	154	Pipeline and inventory levels communication to Field		•					•	•	•		Yes	Yes	Yes
PROC	200	Procurement											Yes	Yes	Yes
PROC	**210**	**Sourcing Strategy**											Yes	Yes	Yes
PROC	211	Establishement of international procurement task force		•	•	•	•	•		•	•	•	Yes	Yes	Yes
PROC	212	"Supplier identification" dedicated communication channels		•	•	•	•	•		•	•	•	Yes	Yes	Yes
PROC	213	Local purchase derogation guideline		•				•		•	•		Yes	Yes	Yes
PROC	**220**	**International Procurement**											Yes	Yes	Yes
PROC	221	Procurement-QA teams restructuring			•	•	•	•		•	•	•	Yes	Yes	Yes
PROC	222	Portfolio reorganization accross ESCs			•	•	•	•		•	•	•	Yes	Yes	Yes
PROC	223	Intersectional purchases to suppliers		•	•	•	•	•		•			Yes	Yes	Yes
PROC	224	Repartition key development for intersectional purchases				•	•	•	•	•	•		Yes	Yes	Yes
PROC	225	ESCs Price/Financial exception validation process			•		•	•		•	•		Yes	Yes	Yes
PROC	226	Intersectional exception validation process				•	•		•		•		Yes	Yes	Yes
PROC	227	Pending order status visbility			•	•	•			•	•		Yes	Yes	Yes
PROC	**230**	**Local Procurement**											Yes	Yes	Yes
PROC	231	Local Suppliers identification	•						•	•	•	•	Yes	Yes	Yes
PROC	232	Intersectional coordination for supplier identification	•				•		•	•	•	•	Yes	Yes	Yes
PROC	233	Validation table adaptation		•					•	•	•		Yes	Yes	Yes
PROC	234	Financial validation process adaptation		•					•	•	•		Yes	Yes	Yes
PROC	235	Securing purchase ahead of quality validation	•	•				•	•	•	•	•	Yes	Yes	Yes
PROC	236	Budget alignement to align with price inflation for critical items		•					•	•	•		Yes	Yes	Yes
PROC	**240**	**Regional Procurement**											Yes	Yes	Yes
PROC	241	Regional Supplier Identifications	•					•		•	•	•	Yes	Yes	Yes
PROC	242	Regional technical support for local procurement	•					•		•	•	•	Yes	Yes	Yes
PROC	243	Regional centers "client mission" protfolio increase	•					•		•	•	•	Yes	Yes	Yes
PROC	244	International purchase direct delivery to regional center			•		•	•		•	•	•	Yes	Yes	Yes
PROC	**250**	**Quality Validation Process**											Yes	Yes	Yes
PROC	251	Quality standard and validation process competency		•	•		•	•	•	•	•	•	Yes	Yes	Yes
PROC	252	International QA validation process		•			•	•			•	•	Yes	Yes	Yes
PROC	253	Adaptation of QA process for suppliers with non-EU conform docs		•			•	•			•	•	Yes	Yes	Yes
PROC	254	Standard local quality validation at OC level		•					•	•	•	•	Yes	Yes	Yes
PROC	255	Quality validation at regional level		•					•	•	•	•	Yes	Yes	Yes
PROC	256	Exception Local Quality Validation by field medical team	•						•	•	•	•	Yes	Yes	Yes
PROC	257	Guideline for quality validation by field teams		•					•	•	•	•	Yes	Yes	Yes
WH	300	Warehousing											Yes	Yes	Yes
WH	**310**	**ESC level**											Yes	Yes	Yes
WH	311	Communication of available preparation capacity			•		•	•			•		Yes	Yes	Yes
WH	312	Exception Prioritatization with OCs		•	•		•	•			•		Yes	Yes	Yes
WH	313	Workaround development for securing pipeline per OCs							•		•		Yes	Yes	Yes
WH	**320**	**Regional level**											Yes	Yes	Yes
WH	321	Increase of regional warehousing activity		•		•	•	•		•	•	•	Yes	Yes	Yes
WH	322	Increase of safety stocks	•			•	•	•		•	•	•	Yes	Yes	Yes
WH	323	Purchase of unallocated stocks of critical items	•			•	•	•		•	•	•	Yes	Yes	Yes
WH	**330**	**Field level**											Yes	Yes	Yes
WH	331	EPREP Usage	•	•									Yes	Yes	Yes
WH	332	Months of inventory increae, storage increase	•					•		•	•	•	Yes	Yes	Yes
TRANSP	400	Transport											Yes	Yes	Yes
TRANSP	**410**	**Idenfication of available means of transport**											Yes	Yes	Yes
TRANSP	411	New transporter identification			•	•	•	•		•	•		Yes	Yes	Yes
TRANSP	412	Inter-ESCs collaboration for transport identification			•	•	•	•		•	•		Yes	Yes	Yes
TRANSP	413	Dedicated intersectional communication channels definition			•	•	•	•		•	•		Yes	Yes	Yes
TRANSP	**420**	**Inbound Transport**											Yes	Yes	Yes
TRANSP	421	New transporter idenfication			•	•	•	•		•	•		Yes	Yes	Yes
TRANSP	422	Collaboration for Inbound flows			•		•	•		•	•		Yes	Yes	Yes
TRANSP	423	Freight forwarding to other ESCs			•		•	•	•	•	•		Yes	Yes	Yes
TRANSP	424	Cross-docking through Dubaï			•	•	•	•		•	•		Yes	Yes	Yes
TRANSP	**430**	**Outbound Transort**											Yes	Yes	Yes
TRANSP	431	EU Export regulation compliance			•			•	•		•		Yes	Yes	Yes
TRANSP	432	Collaboration with WFP and ECHO for flights			•		•	•		•	•		Yes	Yes	Yes
TRANSP	433	Routing optimization dur to personnel restriction			•			•		•	•		Yes	Yes	Yes
TRANSP	434	Validation of freight price estimate	•						•	•	•		Yes	Yes	Yes
ENABLER	500	Enablers											Yes	Yes	Yes
ENABLER	**510**	**Communication**											Yes	Yes	Yes
ENABLER	511	Development of internal cross-department platforms		•	•		•	•	•		•		Yes	Yes	Yes
ENABLER	512	Developemnt of intersectional platforms		•	•	•	•	•	•		•		Yes	Yes	Yes
ENABLER	513	Development of sharepoint pages		•	•	•	•	•	•		•		Yes	Yes	Yes
ENABLER	**520**	**Visibility**											Yes	Yes	Yes
ENABLER	521	Improved collection of field data	•	•				•		•	•		Yes	Yes	Yes
ENABLER	522	Development of tools per OC for increased visbility		•			•	•		•	•		Yes	Yes	Yes
ENABLER	523	Development of intersectional tools for increased visbility		•	•	•	•	•		•	•		Yes	Yes	Yes
ENABLER	**530**	**Software Interoperability**											Yes	Yes	Yes
ENABLER	531	Creation of work-around at regional platform levels		•	•			•	•	•	•		Yes	Yes	Yes
ENABLER	532	Creation of work-around for field collaboration	•					•	•	•	•		Yes	Yes	Yes
ENABLER	**540**	**Workload Capacity**											Yes	Yes	Yes
ENABLER	541	Stand-by of non-essential activity		•	•	•		•	•				Yes	Yes	Yes

APPENDIX 5: DELPHI QUESTIONNAIRE 2

Dear All,

Thank you for attending the first iteration of the Delphi yesterday.
Please find here under the instructions to fill in the following taps, in oder to prepare the second round and adjust the analysis to your needs.
Thank you in advance for your time,
Best Regards,
The HUMLOG Insitute Team

Instructions

Please use the following process to evaluate each level of the frameworks.
Please evaluate the list of processes that were updated/ changed/ added for the Covid-19 response, and rank them in line of their relevance, importance and accuracy **as part of the lessons learned**

A. Input you perspective on each item relevance, importance and accuracy.
B. Please add your comments, concerns and insights to the comments colums of the process tabs when necessary.
A. Rate which areas of recommendation should be prioritized for operational recomendations by the reseach team.
D. Please add your comments, concerns and insights to the comments colums of the recommendation tabs when necessary.

**Relevance: of the updated/changed/added process to the lessons learned**	
**YES**	- Relevant to the analysis and the lessions learned<br>- Must/Should be included
**NO**	- Insignificantly relevant or No relevance<br>- Should be removed

**Accuracy: of the wording of the updated/changed/added process to the lessons learned**	
**YES**	- All necessary information is included and underlying concept for the item is easy to understand<br>or Minor information missing but does not substantially affect understanding of underlying item concept<br>- All terms used are widely understood by the majority of the sector, or specific terms are used but does not cause substantial mis-understanding of the item<br>- Wording is in line with rest of framework
**NO**	- Substantial missing information with unclear underlying concept<br>- Item is too easy to confuse with other item(s) with substantial amount of information that is irrelevant to the underlying concept of the item<br>- Unclear terminology not in line with the organization<br>- Requires a re-write

PROCESS	#	LESSONS LEARNED	RELEVANCE	ACCURACY	IMPORTANCE	COMMENTS
NEEDS	100	Needs Assessment and Planning				
NEEDS	170	Success Factors				
NEEDS	171	Prioritization of needs and urgency levels definition	Yes	Yes	Yes	
NEEDS	172	Centralization of needs assessment at OCs HQ	Yes	Yes	Yes	
NEEDS	173	Integration Strategy of OCs HQ and ESCs	Yes	Yes	Yes	
NEEDS	174	Field Inventory Visibility (when already in place)	Yes	Yes	Yes	
NEEDS	175	Emergency order guideline swift implementation	Yes	Yes	Yes	
NEEDS	176	ESC expertise and reconfiguration potential	Yes	Yes	Yes	
NEEDS	177	Consideration of alternatives product and clear identification of items	Yes	Yes	Yes	
NEEDS	180	Areas of Improvement				
NEEDS	181	Consistency in the strategy of centralization of needs assessment at HQs	Yes	Yes	Yes	
NEEDS	182	Need for clear and separated tactical and strategic governance	Yes	Yes	Yes	
NEEDS	183	Clear governance on communication channels, decision-making and reaching intersectional agreement	Yes	Yes	Yes	
NEEDS	184	Invest in clear guidelines, and invest in consistency across updated guidelines	Yes	Yes	Yes	
NEEDS	185	Field Inventory Visibility (when didn't exist or was insufficient)	Yes	Yes	Yes	
NEEDS	186	When process update needs to align the communication channel	Yes	Yes	Yes	
NEEDS	190	Recommendations				
NEEDS	191	Emergency Needs Assessment Guidelines	Yes	Yes	Yes	
NEEDS	192	EPREP Scenarios with Market Assessments	Yes	Yes	Yes	
NEEDS	193	Forecast and Information Needs Alignment Intersectionnally	Yes	Yes	Yes	
PROC	200	Procurement				
PROC	270	Success Factors				
PROC	271	International Procurement Task-force implementation	Yes	Yes	Yes	
PROC	272	Optimization of resources at knowledge and expertise centers at the ESCs level	Yes	Yes	Yes	
PROC	273	ESCs' autonomy regarding risk management capacity	Yes	Yes	Yes	
PROC	274	Local purchase derogation guidelines and consistent strategy (for some OCs)	Yes	Yes	Yes	
PROC	275	Swift implementation and adaptability of emergency validation tables	Yes	Yes	Yes	
PROC	276	Field teams' reactivity and adaptability	Yes	Yes	Yes	
PROC	277	Intersectional field collaboration (when happened)	Yes	Yes	Yes	
PROC	278	Adaptability when exceptional bottleneck identified (in some cases)	Yes	Yes	Yes	
PROC	280	Areas of Improvement				
PROC	281	Adaptability when exceptional bottleneck identified (in some cases)	Yes	Yes	Yes	
PROC	282	Inclusion of regional and local procurement as part of the overall emergency procurement strategy	Yes	Yes	Yes	
PROC	283	Resources optimization at field level	Yes	Yes	Yes	
PROC	284	Consistency of local procurement derogation strategy	Yes	Yes	Yes	
PROC	285	Optimization of existing regional platforms capacity	Yes	Yes	Yes	
PROC	286	Diversify the supplier portfolio	Yes	Yes	Yes	
PROC	287	Establish crisis repartition key across OCs	Yes	Yes	Yes	
PROC	288	Education to all MSF members regarding procurement stakes	Yes	Yes	Yes	
PROC	289	Alignment of quality standards and emergency quality validatio	Yes	Yes	Yes	
PROC	290	Recommendations				
PROC	291	EPREP Scenarios with Market Assessments	Yes	Yes	Yes	
PROC	292	Emergency Validation Intersectional Alignment	Yes	Yes	Yes	
PROC	293	Medical Local Procurement Process Revision	Yes	Yes	Yes	
PROC	294	Local Quality Validation Process Support	Yes	Yes	Yes	
PROC	295	International Suppliers Portfolio Diversification	Yes	Yes	Yes	
PROC	296	Supply Chain Network Design – Regionalization	Yes	Yes	Yes	
PROC	297	Intersectional IO Quality Assessment	Yes	Yes	Yes	
PROC	298	Local supplier database	Yes	Yes	Yes	
WH	300	Warehousing				
WH	370	Success Factors				
WH	371	High inventory in the field to maintain regular activities	Yes	Yes	Yes	
WH	372	High expertise at ESC level	Yes	Yes	Yes	
WH	373	Good initiatives at regional level	Yes	Yes	Yes	
WH	374	Good initiatives and collaboration field level	Yes	Yes	Yes	
WH	380	Areas of Improvement				
WH	381	Integrate regional platforms within the global strategy	Yes	Yes	Yes	
WH	382	Increase centralized visibility of regional inventory	Yes	Yes	Yes	
WH	390	Recommendations				
WH	391	On-hand inventory for regular activities	Yes	Yes	Yes	
TRANSP	400	International Transport				
TRANSP	470	Success Factors				
TRANSP	471	ESCs transport teams' expertise	Yes	Yes	Yes	
TRANSP	472	Collaboration with WFP and ECHO flights	Yes	Yes	Yes	
TRANSP	473	Cross-ESCs collaboration	Yes	Yes	Yes	
TRANSP	480	Areas of Improvement				
TRANSP	481	Consolidation of transport from ESCs to field	Yes	Yes	Yes	
TRANSP	482	Empowerment of transport team for final calls	Yes	Yes	Yes	
TRANSP	483	Regional deliveries	Yes	Yes	Yes	
TRANSP	490	Recommendations				
TRANSP	491	Transport consolidation process	Yes	Yes	Yes	
TRANSP	492	Transport consolidation strategy	Yes	Yes	Yes	
TRANSP	493	Regional delivery from suppliers	Yes	Yes	Yes	
ENABLERS	500	Enablers				
ENABLERS	570	Success Factors				
ENABLERS	571	New centralized communication channels	Yes	Yes	Yes	
ENABLERS	572	Development of relevant platforms	Yes	Yes	Yes	
ENABLERS	573	Field data collection	Yes	Yes	Yes	
ENABLERS	574	Increased visibility	Yes	Yes	Yes	
ENABLERS	575	Ordering tool workaround for interoperability	Yes	Yes	Yes	
ENABLERS	576	Workaround for intersectional collaboration	Yes	Yes	Yes	
ENABLERS	577	ESCs and OCs workload reconfiguration	Yes	Yes	Yes	
ENABLERS	580	Areas of Improvement				
ENABLERS	581	Internal OC communication channels	Yes	Yes	Yes	
ENABLERS	582	Intersectional communication channels	Yes	Yes	Yes	
ENABLERS	583	Field communication	Yes	Yes	Yes	
ENABLERS	584	Platforms ToR clarification, governance and communication	Yes	Yes	Yes	
ENABLERS	585	Alignment of visibility tools objectives	Yes	Yes	Yes	
ENABLERS	586	Ordering tools interoperability	Yes	Yes	Yes	
ENABLERS	587	ERP interoperability	Yes	Yes	Yes	
ENABLERS	588	Field resources optimization strategy	Yes	Yes	Yes	
ENABLERS	590	Recommendations				
ENABLERS	591	Lessons learned from visibility tools developed	Yes	Yes	Yes	
ENABLERS	592	Lessons learned from platforms established	Yes	Yes	Yes	
ENABLERS	593	Lessons learned from communication channels established	Yes	Yes	Yes	
ENABLERS	594	Skeleton teams and collaboration set-up	Yes	Yes	Yes	
ENABLERS	595	Crisis communication strategy	Yes	Yes	Yes	
ENABLERS	596	Closer inter-department collaboration	Yes	Yes	Yes	
ENABLERS	597	Invest in field workload optimization	Yes	Yes	Yes	
ENABLERS	598	Increase intersectional visibility	Yes	Yes	Yes	
ENABLERS	599	Tools interoperability	Yes	Yes	Yes	

APPENDIX 6 : FIELD QUESTIONNAIRE

MSF intersectional Supply Chain covid-19 response analysis for all regular missions - EN

This questionnaire is conducted by the HUMLOG Institute, from Hanklen School of Economics in Helsinki for all MSF sections. The HUMLOG Institute is conducting an external analysis to identify the strength and optimization possibilities learned from the covid-19 pandemic impact on the Supply Chain.
We ensure the anonymity of all answers, and only aggregated results will be communicated to MSF management.
This questionnaire will take 15 to 20 minutes.
The more comment, the better the analysis and the more chances to induce positive change.

* Obligatoire

1. With which OC were you working from 02/2020 - 08/2020 ? *
   - OCA
   - OCB
   - OCBA
   - OCG
   - OCP

2. In which country were you working from 02/2020 - 08/2020 (if several countries select the main one) *

   Sélectionnez votre réponse

[illegible]

3. To which team / department / unit were you affiliated from 02/2020 - 08/2020 ? *
   - Supply
   - Logistic
   - Med
   - Ops
   - Other

4. During the covid-19 pandemic, which of the following cases applied to your missions *
   - Maintaining of all regular activities
   - Interruption of some of the regular activities
   - Scaling up of regular activities to include additional influx of patients
   - Adaptation of regular activities to align with covid-19 constraints
   - Opening of non-covid related activities
   - Opening of covid-related activities (non emergency)
   - Opening of covid-19 emergency activities
   - Closing of some activities

5. Which Supply Chain Processes were impacted / had to be adapted during the covid-19 pandemic from 02/2020 - 08/2020 ? *
   - Needs assessment
   - International Orders Placement

- ☐ Prioritization of Orders
- ☐ International Pipeline and Visibility
- ☐ Sourcing Strategy
- ☐ International Procurement
- ☐ Regional Procurement
- ☐ Local Procurement
- ☐ Quality Validation Process
- ☐ Inventory Management
- ☐ International Transport
- ☐ Local Transport
- ☐ Communication with HQ
- ☐ Communication with ESC
- ☐ Intersectional Collaboration
- ☐ Workload adjustment

6. Your perception of the impact of the pandemic on the Needs Assessment Process: *

	Strongly Agree	Agree	Disagree	Strongly disagree	Non Applicable
The needs for regular activities and covid-19 regulations was well estimated early on.	○	○	○	○	○
Regular needs assessment process were adapted to the pandemic.	○	○	○	○	○
The implementation of new needs calculation method was done quickly.	○	○	○	○	○
The new needs calculation method was appropriate.	○	○	○	○	○
The HQ guidelines for needs assessment were clear and sufficient.	○	○	○	○	○
The intersection guidelines were clear and sufficient.	○	○	○	○	○
HQ modified the needs you estimated.	○	○	○	○	○
The HQ review of your need was helpful	○	○	○	○	○
The HQ review of your need was conducted in a timely manner.	○	○	○	○	○
The order validation process was adapted to the emergency.	○	○	○	○	○
The order validation process adaptation was helpful for the field.	○	○	○	○	○
The prioritization of international regular orders was in line with the field needs.	○	○	○	○	○
You contributed sufficiently in order prioritization.	○	○	○	○	○
Visibility of inventory and pipeline within your mission was increase.	○	○	○	○	○
Visibility of inventory and pipelines across the supply chain was increased.	○	○	○	○	○
Visibility of inventory and pipepilenes across the supply chain was sufficient.	○	○	○	○	○

7. Do you have any specific comment regarding needs assessment during covid-19 ? eg. Key supportive elements, bottlenecks, problems ? (non mandatory)

Entrez votre réponse

8. Your perception of the impact of the pandemic on the Procurement Process:
*

	Strongly Agree	Agree	Disagree	Strongly Disagree	Non Applicable
International Procurement of regular orders was maintained.	○	○	○	○	○
International Procurement of covid-19 items was successful.	○	○	○	○	○
International Procurement of covid-19 items was sufficient.	○	○	○	○	○
Local Procurement was necessary for covid-19 items.	○	○	○	○	○
Local Procurement was necessary to maintain regular activities.	○	○	○	○	○
The switch from international to local procurement was quickly conducted.	○	○	○	○	○
The instruction to switch to local procurement was advised early enough.	○	○	○	○	○
HQ provided sufficient support to switch from international to local procurement.	○	○	○	○	○
HQ validation process was adapted to fit local procurement needs.	○	○	○	○	○
Budget was adapted to align with additional covid-19 items purchase and price inflation.	○	○	○	○	○
Lack of available budget was a bottleneck for local purchase	○	○	○	○	○
Local medical purchase was conducted before in the mission.	○	○	○	○	○
The knowledge of the market was sufficient to quickly switch to local purchase.	○	○	○	○	○
Relaxed validation process was adapted to the situation.	○	○	○	○	○
The switch to local procurement was positive.	○	○	○	○	○
Regional procurement was in-place and helpful.	○	○	○	○	○
Regional procurement was developed and helpful.	○	○	○	○	○
Regional procurement was developed but problematic.	○	○	○	○	○
Regional platform provided relevant technical support.	○	○	○	○	○
The local procurement					

[illegible] process is adapted for emergencies.	○	○	○	○	○

9. Do you have any specific comment regarding procurement during covid-19 ? eg. Key supportive elements, bottlenecks, problems ? (non mandatory)

Entrez votre réponse

10. Your perception of the impact of the pandemic on the Local Quality Validation Process (Relaxed procedure for quality validation by the field for local procurement) OPTIONNAL:

	Strongly Agree	Agree	Disagree	Strongly disagree	Non Applicable
Local medical quality validation was problematic during the covid-19 pandemic.	○	○	○	○	○
Knowledge for local medical quality validation was already existing in the mission.	○	○	○	○	○
Knowledge for local medical quality validation was already existing at regional level.	○	○	○	○	○
Guidelines to gain knowledge on local quality validation were clear enough.	○	○	○	○	○
Local Quality Validation allowed for items of similar quality as the one sources internationally.	○	○	○	○	○
Intersectional efforts were conducted locally for supplier and quality validation.	○	○	○	○	○
Local Quality validation was maintained at HQ level.	○	○	○	○	○
Local Quality validation maintained at HQ level was fast enough.	○	○	○	○	○
HQ support was sufficient for local quality validation.	○	○	○	○	○
Regional platforms assisted in quality validation.	○	○	○	○	○
Quality standards and validation were consistent across missions from different OCs within your country.	○	○	○	○	○
The quality validation process is good enough for emergency local procurement.	○	○	○	○	○
Additional quality validation training is needed in your mission.	○	○	○	○	○
Local quality validation is riskier than quality validation at HQ level.	○	○	○	○	○
Local quality validation is riskier than international procurement.	○	○	○	○	○

11. Do you have any specific comment regarding Local Quality Validation during covid-19 ? eg. Key supportive elements, bottlenecks, problems ? (non mandatory)

Entrez votre réponse

12. Your perception of the impact of the pandemic on the International Transport of Goods Process: *

	Strongly Agree	Agree	Disagree	Strongly disagree	Non Applicable
International Transport of goods was a key problem during the pandemic.	○	○	○	○	○
International Transport availability caused critical delay in regular orders.	○	○	○	○	○
The increase of transport price was a cause of delay due to lack of mission budget.	○	○	○	○	○
ESC handled most of the complexity due to international transport during the pandemic.	○	○	○	○	○
The mission handled most of the complexity due to international transport during the pandemic.	○	○	○	○	○
HQ was supportive with the complexity due to international transport during the pandemic.	○	○	○	○	○
Local intersectional initiatives helped with international transport issues.	○	○	○	○	○
Regional initiatives helped with international transport issues.	○	○	○	○	○
Collaboration with other NGOs/UN helped with international transport issues.	○	○	○	○	○

13. Do you have any specific comment regarding international transport during covid-19 ? eg. Key supportive elements, bottlenecks, problems ? (non mandatory)

Entrez votre réponse

14. Your perception of the impact of the pandemic on the Inventory Management Process: *

	Strongly Agree	Agree	Disagree	Strongly disagree	Non Applicable
Safety stocks levels contributed to manage disruptions due to covid	○	○	○	○	○
Overstock contributed to manage disruptions due to covid	○	○	○	○	○
EPREP Stocks contributed to manage disruptions due to covid	○	○	○	○	○
Intersection loans and donations contributed to manage disruptions due to covid	○	○	○	○	○

15. Do you have any specific comment regarding inventory management during covid-19 ? eg. Key supportive elements, bottlenecks, problems ? (non mandatory)

Entrez votre réponse

16. Your perception of the impact of the pandemic on the Local Transport of Goods Process: *

- ☐ Local transport was a key bottleneck during the pandemic
- ☐ Local transport was a complexity but not a bottleneck
- ☐ Local transport didn't change much during the pandemic
- ☐ Local transport was not an issue during the pandemic

17. Your perception of the Communication with Headquarters during the pandemic: *

	Strongly Agree	Agree	Disagree	Strongly disagree	Non Applicable
More information were requested by HQ during the pandemic.	○	○	○	○	○
Information requested by HQ during the pandemic increased the workload.	○	○	○	○	○
Communication from HQ during the pandemic increased.	○	○	○	○	○
Communication from HQ was sufficient.	○	○	○	○	○
Communication from HQ provided clear understanding of the constraints across the Supply Chain.	○	○	○	○	○
Communication from HQ provided clear visibility across the Supply Chain.	○	○	○	○	○
[illegible]					
Communication from HQ provided helpful guidance.	○	○	○	○	○
Communication from HQ supported intersectional collaboration.	○	○	○	○	○
HQ answers time was sufficient to resolve questions in a timely manner.	○	○	○	○	○

18. Your perception of the communication with European Supplier Centers ESCs (MSF Logistique, MSF Supply or APU) *

	Strongly Agree	Agree	Disagree	Strongly disagree	Non Applicable
Communication with ESC during the pandemic increased.	○	○	○	○	○
Communication from ESCs was sufficient.	○	○	○	○	○
Communication from ESCs provided clear understanding of the constraints across the Supply Chain.	○	○	○	○	○
Communication from ESC provided helpful guidance.	○	○	○	○	○
ESC answers time was sufficient to resolve questions in a timely manner.	○	○	○	○	○

19. Do you have any specific comment regarding communication with HQ or ESC ? eg. Key supportive elements, bottlenecks, problems ? (non mandatory)

Entrez votre réponse

Entrez votre réponse

20. Your perception of the impact of MSF governance during the pandemic : *

	Strongly Agree	Agree	Disagree	Strongly Disagree	Non Applicable
Missions had sufficient mechanism in place to adapt to emergency situation.	○	○	○	○	○
HQ instruction to activate emergency mechanism were quick enough	○	○	○	○	○
HQ derogated sufficient validation power for missions to react quickly.	○	○	○	○	○
HQ addressed the proper issues faced by your mission	○	○	○	○	○

21. Do you have any specific comment regarding the governance ? eg. Key supportive elements, bottlenecks, problems ? (non mandatory)

Entrez votre réponse

22. Your perception of your workload during the pandemic : *

	Strongly Agree	Agree	Disagree	Strongly disagree
Your workload increased a lot during the pandemic.	○	○	○	○
You were able to put some activities on standby.	○	○	○	○
You were able to hire additional staff to handle the workload.	○	○	○	○
You had to increase overtime to handle the workload.	○	○	○	○

23. Thank you very much for your time ! Please feel free to add additional comments.

Entrez votre réponse

Envoyer

Ne communiquez jamais votre mot de passe. Signaler un abus

Ce contenu est créé par le propriétaire du formulaire. Les données que vous soumettez sont envoyées au propriétaire du formulaire. Microsoft n'est pas responsable des pratiques de confidentialité ou de sécurité de ses clients, y compris celles de ce propriétaire de formulaire. Ne donnez jamais votre mot de passe.

Avec Microsoft Forms |
Le propriétaire de ce formulaire n'a pas fourni de déclaration de confidentialité quant à la façon dont il utilisera vos données de réponse. Ne fournissez pas d'informations personnelles ou sensibles.
| Conditions d'utilisation | Accessibilité

APPENDIX 7: SABRI'S (2019) COLLABORATIVE METHODOLOGY

Sabri, Y., Zarei, M.H., Harland, C. 2019. Using collaborative research methodologies in humanitarian supply chains. *J. Hum. Log. Supply Chain Manag*. **9**(3):371-409.

Collaborative Project Phases	Collaborative Research Features/Elements
1. Forming a collaborative research team of humanitarian logistics practitioners and academics	Forming a project team with representative of all OCs and ESCs, referred to as "MSF Team"
2. Understanding the context and purpose of humanitarian logistics research problem	Co-identification of the rationale and scope: Mitigation strategies Covid-19 pandemic disruptions: process reconfiguration
	Co-identification of a preliminary research question and deciding on the unit of analysis: How process modularity did allowed for disruptions mitigation?
	Researchers are immersed, embedded in the humanitarian field, and they have access to the practitioner's system
	To ensure rigor, involving a non-participatory researcher to monitor and observe the rigor of the entire research process
3. Data collection (by humanitarian logistics and supply chain researchers)	Triangulation of research methods: statistical analysis, content-based analysis, interviews, and Delphi
	Triangulation of data collection from multiple sources: MSF documents and website, international Covid-19 situation report, interviews form field teams from multiple locations and interviews of HQ staff
	Collecting qualitative data : Situation reports, mission strategies, task-forces meeting minutes, observations, focus group discussions, etc…
	Collecting quantitative data : full ERP extraction, order information, stock levels, purchase volumes, lead-times, trasnport data, etc…
	Collecting data in formal meetings, field questionnaire and interviews
	Bi-monthly reflective sessions to discuss and update data collection techniques
4. Practitioner orientation	Practitioners briefed on research tools and methods
	Researchers to prepare and present preliminary analyses: quantitative modeling and qualitative content analysis
	The structured data are communicated to the research team and to the practitioner's personnel

5. Collaborative data analysis	Identifying analysis tools (Excel and Nvivo) and techniques by researchers
	Data are collaboratively analyzed by researchers and practitioners: Delphi iterations
	Triangulation of researchers in the analysis phase
	Establishing a logical chain of evidence by researchers
6. Joint planning for action	Co-identification of what needs to change, and strategies and practices for change management
	Co-developing of recommendations and intervention plans
7. Implementation by humanitarian logistics practitioners with review and evaluation supported by researchers	Practitioners to execute the intervention plan (or to facilitate the implementation with local authorities in the humanitarian field)
	Researchers to ensure that the applicability, re-applicability and transferability conditions are met
	The impact of the implementation to be co-evaluated and co-reviewed by researchers and practitioners
	Joint reflective sessions and co-planning for future action cycles (if needed), which include continuous refinement of the proposed solutions
8. Monitoring of the research by the non- participatory researcher	Monitoring is conducted by a meta-step in this framework: it is facilitated by recruiting a non-participatory researcher who accompanies the research team in all the phases and observes the consistency of the research process and the active participation of all the involved actors
	Monitoring researcher to ensure that rigor conditions are met for any methodology used

APPENDIX 8: SUPPLY CHAIN PROCESSES' MODULAR ARCHITECTURE AT EUROPEAN SUPPLIER CENTERS

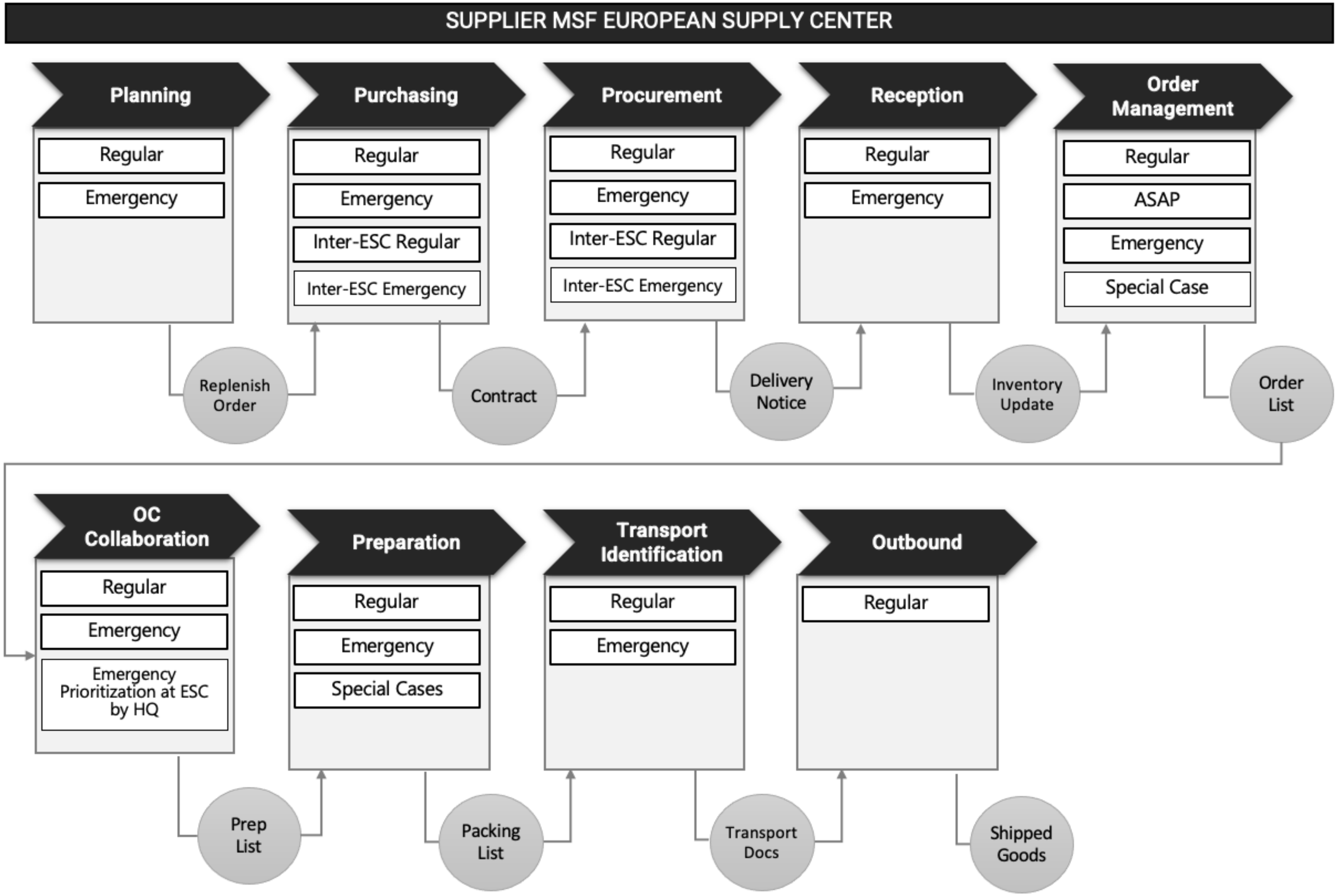

Supplement: Supplementary file 1 — Supporting Information [file POMS-9999-0-s001.pdf]
